# Exploring the Interaction of SV2A with Racetams Using Homology Modelling, Molecular Dynamics and Site-Directed Mutagenesis

**DOI:** 10.1371/journal.pone.0116589

**Published:** 2015-02-18

**Authors:** Joanna Lee, Veronique Daniels, Zara A. Sands, Florence Lebon, Jiye Shi, Philip C. Biggin

**Affiliations:** 1 Structural Bioinformatics and Computational Biochemistry, University of Oxford, South Parks Road, Oxford, OX1 3QU, United Kingdom; 2 UCB Pharma S.A., Chemin du Foriest, B-1420 Braine-l’Alleud, Belgium; Bioinformatics Institute, SINGAPORE

## Abstract

The putative Major Facilitator Superfamily (MFS) transporter, SV2A, is the target for levetiracetam (LEV), which is a successful anti-epileptic drug. Furthermore, SV2A knock out mice display a severe seizure phenotype and die after a few weeks. Despite this, the mode of action of LEV is not known at the molecular level. It would be extremely desirable to understand this more fully in order to aid the design of improved anti-epileptic compounds. Since there is no structure for SV2A, homology modelling can provide insight into the ligand-binding site. However, it is not a trivial process to build such models, since SV2A has low sequence identity to those MFS transporters whose structures are known. A further level of complexity is added by the fact that it is not known which conformational state of the receptor LEV binds to, as multiple conformational states have been inferred by tomography and ligand binding assays or indeed, if binding is exclusive to a single state. Here, we explore models of both the inward and outward facing conformational states of SV2A (according to the alternating access mechanism for MFS transporters). We use a sequence conservation analysis to help guide the homology modelling process and generate the models, which we assess further with Molecular Dynamics (MD). By comparing the MD results in conjunction with docking and simulation of a LEV-analogue used in radioligand binding assays, we were able to suggest further residues that line the binding pocket. These were confirmed experimentally. In particular, mutation of D670 leads to a complete loss of binding. The results shed light on the way LEV analogues may interact with SV2A and may help with the on-going design of improved anti-epileptic compounds.

## Introduction

Epilepsy remains one of the most debilitating neurological disorders and is characterized by recurrent spontaneous seizures [1]. Furthermore, about 30% of patients with epilepsy cannot be adequately treated due to poor efficacy or undesirable side-effects [2]. One of the more successful treatment strategies has revolved around the administration of a compound from a class of compounds collectively known as racetams, which are distinguished by a central pyrrolidine ring system. The only example currently on the market is levetiracetam (LEV; (*S*)-α-ethyl-2-oxo-pyrrolidine acetamide; KEPPRA) which is a second-generation anti-epileptic drug [3]. Studies in rats in the 1990s led to the identification of the target for the action of LEV being the synaptic vesicle protein, SV2A [4]. It has since been confirmed as the target for related racetam compounds in both rat and human brains [5]. Although the mode of action of LEV is not known at the molecular level, it appears to have a different activity profile compared to many anti-epileptic drugs and thus may act via a different pathway, which in turn may account for fewer adverse side-effects when compared to many existing anti-epileptic compounds [6]. Thus there is considerable interest in understanding its interaction with SV2A.

SV2A is an integral membrane protein found in both synaptic and dense-core vesicles [7]. It is one of three SV2 isoforms that are distributed widely across the brain and has been found to be necessary for Ca^2+^-dependent vesicular neurotransmitter release [8,9]. It has been implicated in the formation of the SNARE complex [9], with a proposed binding site for synaptotagmin in the amino terminal domain of SV2A. There is also a putative ATP binding site [10], though SV2A has not been proven to affect ATP uptake in synaptic vesicles [11]. Despite intense effort, the actual physiological function of SV2A remains unclear, though recently it has been shown to be capable of transporting galactose at least when expressed in hexose transport-deficient EBY.VW4000 yeast cells [12].

Knock out mice die within 3 weeks of birth, suffering seizures within 7 days [13], which correlates with the first expression of SV2A [14]. Furthermore, in knock-out models, neurons exhibited sustained increase in Ca^2+^ dependent synaptic transmission when two or more action potentials were triggered in quick succession [15]. Although Wan *et al*. [16] have shown that retinal neurons from SV2B knockout mice exhibit apparent changes in cytoplasmic calcium at the presynaptic terminal, the precise role or relevance of SV2 proteins in calcium homeostasis remains rather unclear at the present time. Kindling experiments, where repeated stimulation induces seizures that are proposed to mimic partial onset epilepsy, have indicated an up-regulation of SV2A in rat models [17]. However, other experiments have shown that SV2A expression decreases in chronically epileptic animals [18] and in patients with temporal lobe epilepsy [19]. This experimental data points towards a key role in synaptic maturation, though the mechanism for that remains unclear.

By sequence homology, SV2A has been described as a member of the Major Facilitator Superfamily (MFS) of transporters [20–22]. Structural evidence for this came from protein tomography experiments where two major conformations could be inferred [23]. Furthermore, LEV binding did not cause large-scale conformation changes or appear to lock the protein in a specific conformational state. Ligand-binding assays have also suggested that the conformational state of SV2A can be modulated in an allosteric fashion [24]. Taken together, these data demonstrate that the SV2A protein is likely to be highly dynamic and adopt many conformational states.

Although there is no structure for SV2A, it has been postulated, through remote sequence relationships, to resemble the architecture found in the MFS clan of transporters. However, the sequence identity to known examples of this family is very low (16% and 15% for GlpT and FucP respectively). If indeed it is a true member of the MFS family then one might expect it to be involved in the uptake of a key metabolite, which would be transported via the alternating access mechanism [25]. Work by Shi et al. [26] determined 13 residues important for binding the racetam, ucb 30889, which were chosen according to their alignment to functionally relevant residues in LacY. Here we extend this work further by considering two alternative conformational states based on two additional templates: FucP as a template for the outward-open state and GlpT as an additional (to LacY) model of the inward-open state. In this terminology, inward refers to the cytosol, thus as SV2A is a synaptic vesicle membrane protein, an outward-facing state means the binding site would be exposed to the inside of the vesicle. On the basis of *in-silico* modelling and molecular dynamics simulations, we were able to suggest additional residues that line the binding pocket for ucb 30889. These predictions were subsequently confirmed by site-directed mutagenesis in conjunction with binding affinity assays. Taken in conjunction with previous results, the outward-open models (i.e. with the putative binding site exposed to the interior of the vesicle) tend to give better agreement with site-directed mutagenesis experiments.

## Materials and Methods

### Sequence Analysis and Model Building

Transmembrane (TM) predictions for the 12 potential TM helices of SV2A were made with SOSUI [27], HMMTop [28], JPRED [29] and PSIPRED [30]. A final consensus TM prediction was made manually (see S1 Fig.). As we do not know which conformational state of SV2A compounds like levetiracetam (LEV) bind to, we explored two distinct models corresponding to an inward-open and outward-open state. The FucP structure (PDB: 3O7P [31]) was used as the template for the outward-open state and GlpT (PDB: 1PW4 [32]) was used as the template for the inward-open state. Initial structural alignments based on the consensus predictions were manually adjusted. These alignments were used to generate homology models with Modeller 9.10 [33]. In the case where the model was predicted to be helical but the template did not have helical structure, restraints were applied within Modeller to ensure helicity. As the focus here was on the racetam-binding site within the TM region, we did not attempt to make structural predictions for TMH-connecting regions and modeled them as loops within Modeller. 100 models were generated in each case and the model with the best DOPE score [34] was selected.

**Fig 1:**
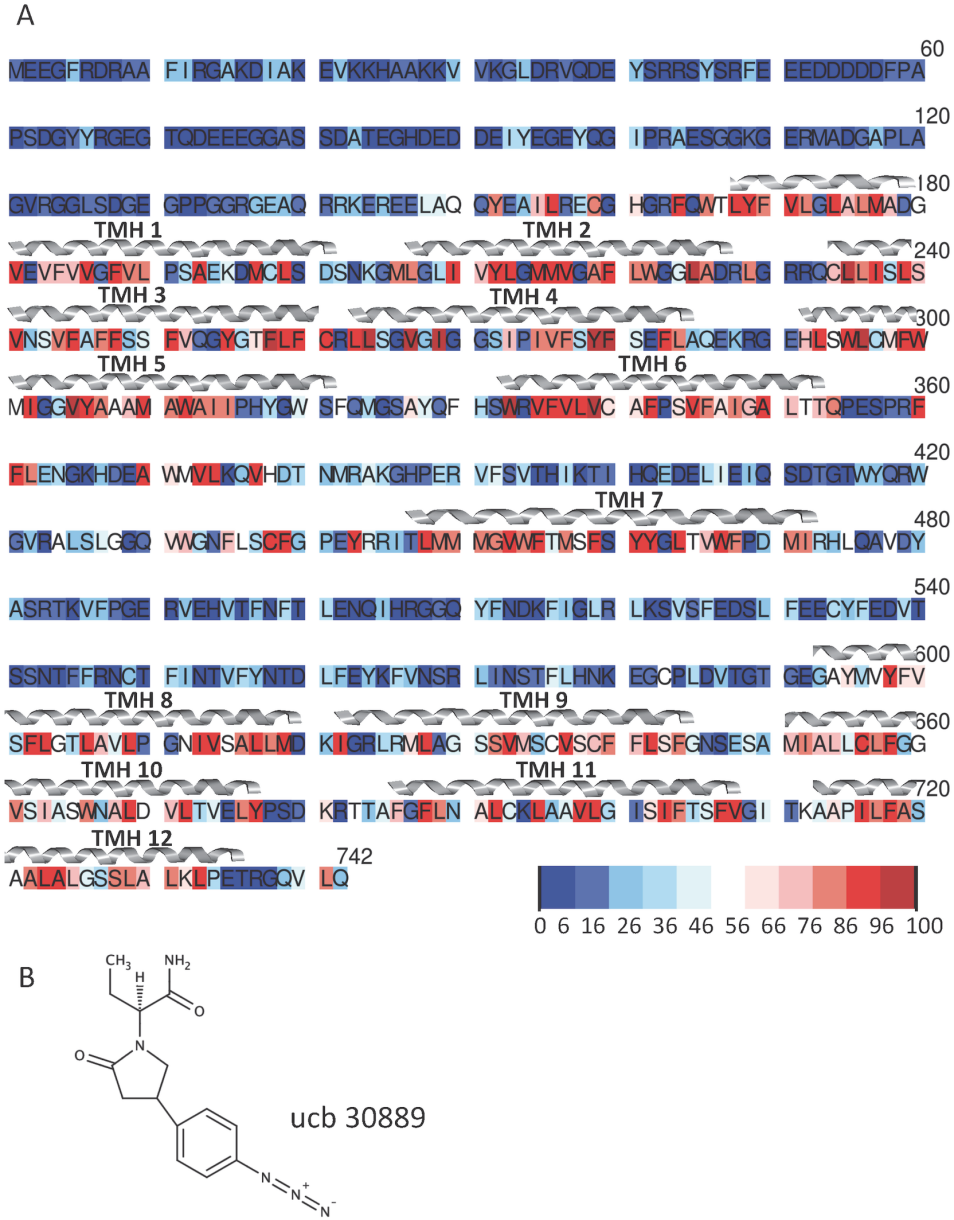
(A) Conservation pattern of residues (M, A, V, I, L, C, Y, W and F) as ascertained by an alignment of 758 sequences form a BLAST search against rat SV2A . The degree of conservation is coloured from blue to red as a function of percentage. The position of the TM helices as predicted from the consensus prediction (S1 Fig.) are indicated. (B) Chemical structure of ucb 30889, a commonly used radio-ligand that is an analogue of LEV.

To refine these models further we used conservation data from a sequence alignment. A BLAST [35] search with SV2A_RAT (Uniprot: Q02563) as the query sequence returned 758 sequences with E < 10^–30^ and an alignment was constructed using CLUSTAL Omega [36]. The conserved sites were determined using in-house R scripts to produce a heat map: blue—red corresponding to 0–100% conservation. More usefully, for the initial refinement of the transmembrane helices, we generated heat-maps for the degree of hydrophobic conservation which we determined by computing the occurrence of residues M, A, V, I, L, C, Y, F and W (Fig. 1), as has been previously demonstrated for class A G-protein Coupled Receptors [37].

We then examined the initial models and used the conservation analysis to refine the structural alignment. The final alignment is given in Supporting Information (S2 Fig.). Models were then constructed using Modeller as before and additionally assessed in terms of their QMEAN score [38] to provide an absolute measure of the structural quality that can be compared to known structures.

**Molecular Dynamics**. The final models were embedded in a 1-palmitoyl-2-oleoyl-sn-glycero-3-phosphocholine (POPC) bilayer using the *g_membed* [39] feature of GROMACS and an energy minimization with a steepest descent algorithm until convergence with a force tolerance of 0.239 kcal mol^−1^ Å^−1^ was performed. Sodium and chloride ions were then added to the systems to a concentration of 150 mM followed by as restrained MD run whereby all heavy atoms were restrained by a harmonic potential of 2.39 kcal mol^−1^ Å^−2^ for 1 ns. Finally, 80 ns of production runs were performed on three repeats that differed in their initial velocities only. As an additional check of the simulations we analyzed the area per lipid for all simulations and observed that there was no significant difference compared to t = 0 or between runs. The areas per lipid (in Å^2^) for the Inward-apo, Inward-ucb 30889, Outward-apo and Outward-ucb 30889 simulations were 65.58 ± 0.82, 68.63 ± 0.74, 68.21 ± 0.71 and 67.49 ± 0.71 respectively. Simulations were performed for inward and outward models both in the apo state and in complex with the radioligand (used in previous functional assays) ucb 30889 as summarized in Table 1. MD simulations were carried out with GROMACS v4.5.4 [40] using the OPLS-AA [41,42] force-field and TIP3P water molecules [43]. Production simulations were performed in an NPT ensemble maintained at 323 K and 1 bar pressure. The integration time step was set as 2 fs and a stochastic dynamics integrator [44] was used. Long-range electrostatics were calculated using the particle mesh Ewald (PME) method [45] with a 14 Å cut-off and 1 Å space grid. The Lennard-Jones potential used a cut-off of 9 Å, with a switch at 8 Å. The LINCS algorithm [46] was used in order to constrain bond lengths in both the lipid molecules and the protein. For the simulations with ucb 30889 bound, the protein coordinates were taken from the apo simulation at 80 ns timestep and Autodock Vina [47] was used to dock the ligand into the cavity to provide the initial starting coordinates for the protein-ligand complex. The docking grid was defined as a rectangle that encompassed the TM cavity between the lipid head groups of the bilayer. The conformation of ligand chosen was that with the best score and energy minimization of the complex was conducted as above, before MD simulation.

**Table 1:** Summary of Simulations.

Name	Description
Inward-apo	Model based on GlpT template
Outward-apo	Model based on FucP template
Inward-ucb 30889	Inward apo model with ucb 30889 docked into the binding pocket
Outward-ucb 30889	Outward apo model with ucb 30889 docked into the binding pocket

Simulations were 80ns in duration and were repeated with 3 different random seeds for the initial velocity assignment.

**Radioligands, drugs and chemicals**. ucb 30889 ((2S)-2-[4-(3-azidophenyl)-2-oxopyrrolidin-1-yl]butanamide) was synthesized at UCB (Braine-l’Alleud, Belgium). [^3^H]ucb 30889 (47 Ci mmol^-1^) was custom labeled by Amersham Biosciences (Roosendaal, The Netherlands). HEK cells and zeocin were purchased from Life Technologies (Merelbeke, Belgium). Phosphate buffered saline (PBS), Dulbecco’s Modified Eagle Medium (DMEM), L-glutamine, trypsin and fetal bovine serum were purchased from Lonza (Verviers, Belgium). Complete protease inhibitor cocktail was purchased from Roche (Vilvoorde, Belgium) and DNAse (Deoxyribonuclease I, Type II from Bovine Pancreas) from Sigma Aldrich (Bornem, Belgium). GF/B filters and GF/C filters were from Brandel (Alpha Biotech Ltd., London, UK) and Pall (Zaventem, Belgium) respectively. All other reagents were of analytical grade and obtained from conventional commercial sources.

**Preparation of membrane proteins from HEK cells**. Human SV2A was cloned from a fetal brain cDNA library as described in Lynch *et al*. [4] SV2A point mutants were synthesized at Genscript and cloned into pcDNA3.1(+) (Life Technologies, Gent, Belgium). Final constructs were verified by sequencing and were expressed using transient transfections in FreeStyleTM 293-F suspension cells using 293fectinTM following the manufacturers instruction (all from Life Technologies). Cells were subcultured in DMEM containing 200 mM of L-glutamine and 100 μg ml^-1^ zeocin, supplemented with 10% fetal bovine serum. The cells were grown in a humidified atmosphere of 5% CO_2_ at 37°C. Confluent cells were pelleted by centrifugation at 1,500 g for 10 min at 4°C. The pellet was washed once with ice cold PBS using the same centrifugation protocol. The resulting pellet was homogenized in a buffer containing 15 mM Tris-HCl, 1 mM EGTA, 0.3 mM EDTA and 2 mM MgCl2 (pH 7.5) supplemented with complete protease inhibitor cocktail Roche. The homogenate was freeze-thawed twice and equilibrated at 25°C followed by a 10 min DNAse (10 U ml^-1^) treatment. Subsequently, the solution was centrifuged for 25 min at 40,000 g and 4°C. Finally, the pellet was resuspended in a buffer containing 20 mM Tris-HCl (pH 7.4) and 250 mM of sucrose at a protein concentration of 5 to 10 mg ml^-1^ and stored in liquid nitrogen.

**Competition binding experiments**. Experiments were performed essentially as described before [5]. For all assays, membrane proteins (10 μg per assay) were incubated for 120 min at 4°C in 0.2 ml of a 50 mM Tris-HCl buffer (pH 7.4) containing 2 mM MgCl_2_. Increasing concentrations of compounds were added in the presence of 5 nM of [^3^H]ucb 30889. At the end of the incubation period, the membrane-bound radioligand was recovered by rapid filtration through GF/B glass fiber filter plates pre-soaked in 0.1% polyethyleneimine (PEI). Plates were washed rapidly with 0.8 ml of ice-cold Tris buffer (pH 7.4); the total washing procedure did not exceed 10 sec. Scintillation cocktail (Ultima Gold MV, Perkin Elmer, Zaventem, Belgium) was added to the filter plates and the radioactivity trapped on the filters was measured using a β-counter.

**Data analysis**. IC_50_ values of competition binding experiments were calculated using computerized nonlinear curve fitting methods (Graphpad Prism 6 software, San Diego, CA), according to the equation of a sigmoidal dose response curve with variable slope.

## Results and Discussion

**Fig 2:**
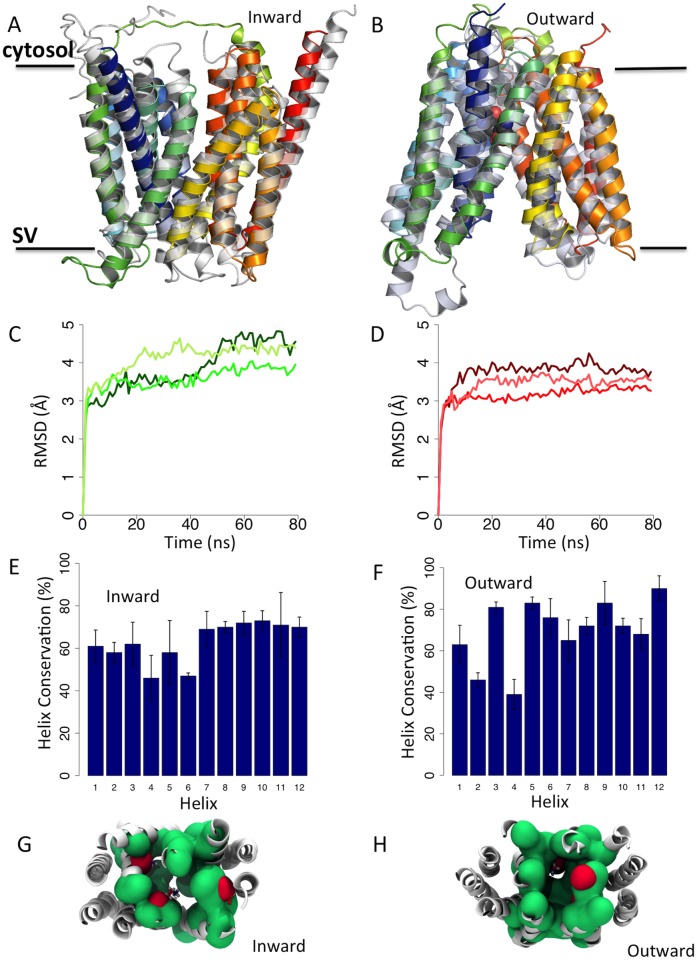
(A) Models of the Inward (based on GlpT template shown in grey) and (B) Outward (based on FucP template shown in grey) SV2A protein. (C) Root mean squared deviation (RMSD) of the Inward-apo (green lines) and (D) the Outward-apo (red lines) simulations (three independent simulations each) over 80 ns. The degree of helix conservation as described by DSSP for each residue in the (E) Inward-apo and (F) Outward-apo models of SV2A. Error bars are the standard deviation (n = 3). (G) A space fill view of the cavity for the Inward-apo and (H) Outward-apo models, with hydrophobic residues coloured green and polar residues shown in red.

Given that SV2A has a low sequence identity to any of the known MFS structures (GlpT has the highest at 16% when aligned to SV2A TM domains: residues 170–375, 445–480, 590–742), and consequently the accuracy of any structural predictions is likely to be low, we attempted to supplement this with multiple sequence comparisons. An alignment of 758 sequences (see Methods) was used to investigate to what extent hydrophobic conservation could be used to suggest TM helix positions. When the conservation of hydrophobic residues is analysed (as shown in Fig. 1), and compared to the consensus TM predictions (S1 Fig.), there is good agreement, thus giving us confidence in the TM predictions. We used this information to structurally align the TM helices of SV2A to FucP and GlpT to provide models of the Outward and Inward (with respect to the cytosol) facing conformations respectively. The alignment was further refined (S2 Fig.) using the hydrophobic conservation patterns, which correspond to buried faces of the helices. The quality of the resulting models (Fig. 2A, B) was assessed with QMEAN where the scores indicated that they sit within the expected range for membrane proteins. The inward-facing model had a score of 0.301 (the range for all 100 models was between 0.330 and 0.401) which when compared to the GlpT template, which has a score of 0.525, was considered reasonable. The outward-facing model had a score of 0.381 (the range for all 100 models was between 0.317–0.388) which when compared to the FucP template with a QMEAN score of 0.512 was also considered reasonable. We also used QMEANclust [48] to assess the confidence of model quality in both models. Unsurprisingly, the loop regions had the highest estimate error (see S3 Fig.).

To explore the conformational stability of both models, we performed MD simulations. The root means square deviation (RMSD) of the TM helix Cα, averaged over three runs for each model, were found to stabilize to 4.29 ± 0.07 Å and 3.57 ± 0.07 Å (Fig. 2C,D) for the Inward-apo and Outward-apo models respectively. A second factor for model validity is the packing of the helices, and to determine this the degree of deviation from an ideal α-helix was calculated (Fig. 2E,F). Since SV2A is a putative transporter that may undergo conformational change as part of its function, a certain amount of structural fluctuation might be expected. In terms of helical character, the apo-systems have greater than 60% conservation of helicity (as defined by DSSP [49]) in all but 3 helices (TMHs 2, 4 and 6) for the Inward-apo and 2 helices (TMHs 2 and 4) for the Outward-apo model, which we take to indicate adequate TMH packing in the models, given that simulations of the templates, GlpT and FucP, maintained helicity in equivalent TM regions. We should note an important caveat at this point and that is that we have performed these simulations in a pure POPC bilayer, and thus at this stage we cannot rule out the specific effects of lipid and protein components that might be found *in vivo*. Nevertheless, these simulations should provide some reassurance that the model is reasonable and compatible with a membrane environment.

We then proceeded to analyze the cavity in the different models. The fluctuations in the volume throughout the simulations were smaller than the differences between models. For example the Inward-apo simulation had a volume of 3843 ± 158 Å^3^ whilst the Inward-ubc 30889 simulation had a mean volume of 3263 ± 111 Å^3^. The outward models had similarly low levels of fluctuation; 2929 ± 45 Å^3^ and 3553 ± 103 Å^3^ for the Outward-apo and Outward-ucb 30889 simulations respectively. These data indicate that on this timescale the models are conformationally stable. The residues lining the cavity are predominately hydrophobic in character (Fig. 2G, H). Further analysis of the conservation of residues within the proposed binding site indicates a conservation of hydrophobicity in this specific region of the cavity. In particular V276, F280, L284 and L296 have hydrophobic conservations between 76 and 96%, despite lower conservations of the particular residue found in each site of SV2A (Table 2) and all of which interact with the docked ligand in both the Inward and Outward models.. This conservation suggests a functional relevance in these positions, tentatively indicating that the endogenous ligand would have some hydrophobic character, especially considering the importance of W300, Y462 and W666 in racetam binding, as determined by Shi et al. [26] all of which display hydrophobic conservation in those sites of 93.9, 86.1 and 82.5% respectively.

**Table 2:** Conservation of residues in an alignment of 758 sequences from a BLAST search of SV2A.

Residue	Conservation (%)	Hydrophobic Conservation (%)
*SDM residues*		
C297	28.4	35.5
W300	83.4	93.9
Y462	83.0	86.1
W666	60.4	82.5
K694	66.4 (R), 23.9 (K)	
*Charged residue in cavity*		
E194	96.6	
*Other residues in cavity*		
W454	61.5	79.8
F688	24.9	88.1
*Hydrophobic pocket*		
V296	61.4	96.0
F280	30.7	80.2
L284	41.8	96.3
L296	40.8	76.3

In addition to the hydrophobic character, there are 3 charged residues within the TM cavity in both models (Table 2), two of which (K694 and E194) are highly conserved. The final residue, D670, has 45% conservation (across the 758 sequences obtained from BLAST; see Methods), though an alignment of just the 24 SV2A sequences indicates that this aspartate is 100% conserved (S4 Fig.) suggesting a role specific to SV2A, rather than a broader function applicable to related proteins. D670 has not been postulated to be involved in ligand-binding before, however its position correlates well with the position of charged residues in other MFS transporters (for example the E135 residue in FucP). The models indicate that D670 is in close proximity to K694 and also to other residues already determined by Shi et al. [26] to impact racetam binding (W300 and Y462). E194 is located towards the cytosolic end of the TM region and so in these models is not expected to form part of the racetam binding site *per se* though its conservation of 97% implies an important but as of yet undefined role.

**Fig 3:**
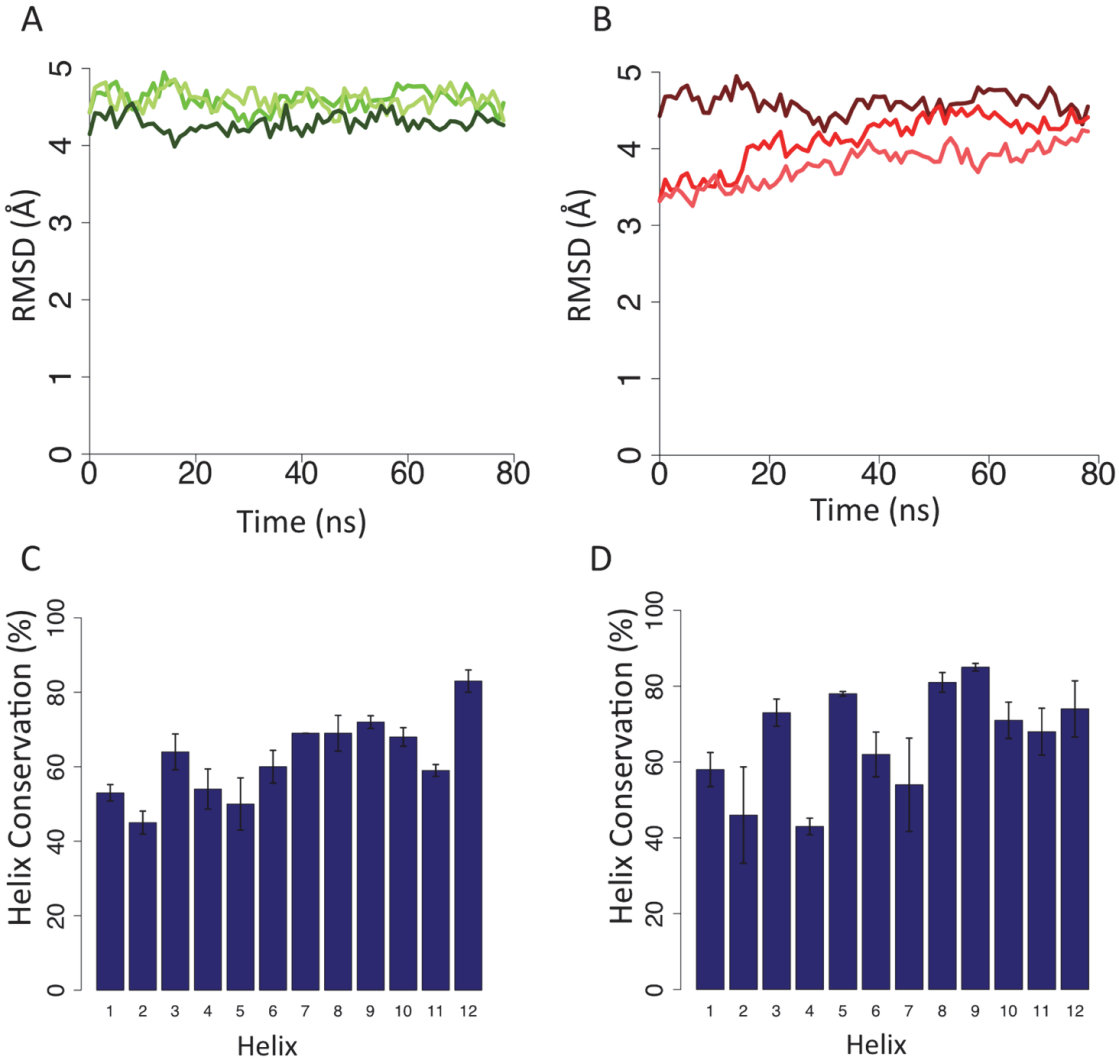
(A) Change in RMSDs from 80 ns of the Inward-ucb 30889 simulation (green) and (B) the Outward-ucb 30889 simulation (red lines) from the initial snapshot from the respective apo simulations . Helix conservation in the (C) Inward-ucb 30889 and (D) Outward-ucb 30889 simulations.

The ligand [^3^H]ucb 30889 (Fig. 1B) has been used in assays on site-directed mutagenesis (SDM) studies reported previously. We docked this compound into the TM cavity of both the inward and outward SV2A models and ran further MD to assess the interaction of the compound with the protein and its influence on the dynamics. RMSD values of the TM Cα atoms show a plateauing (Fig. 3A, B) but at slightly higher values than the apo MD runs at 4.59 ± 0.08 Å (Inward-ucb 30889) and 4.14 ± 0.06 Å (Outward-ucb 30889), suggesting there is some fluctuation in these models after docking the ligand to the apo-system. This observation was supported by the amount of helicity that was preserved in the models. TMHs 1, 2, 4, 5 and 11 are below 60% conservation for the Inward-ucb 30889 run (Fig. 3C) and for the Outward-ucb 30889 run, TMHs 1, 2, 4, 6 and 7 are below 60% (Fig. 3D). All these helices, except TMH 6 line, the cavity, indicating the helices are changing conformation in order to accommodate the ligand. Though the timescale of the simulation is too short to see the large movements associated with the transition between distinct conformational states (expected to be of the order of a few Ångstroms on the basis of structures from other MFS proteins), these fluctuations in the helices could represent the commencement of the protein moving to an occluded state from the open states. However, in order to address that much longer simulation times would be required.

**Fig 4:**
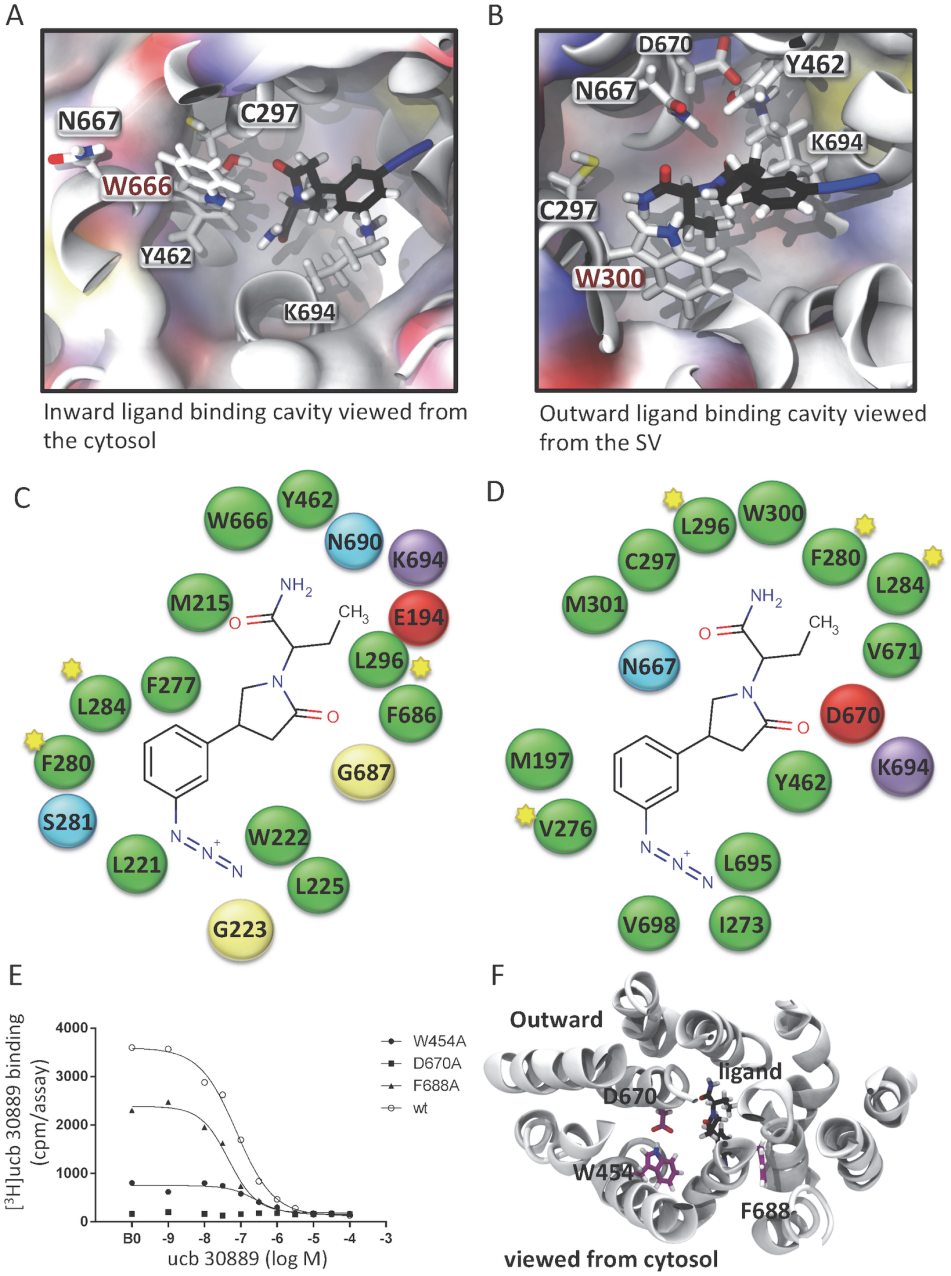
The ligand binding sites in the Inward-apo model of SV2A (A) and the Outward-apo model (B) from simulation (60 ns) . The ligand (black stick) was docked to a snapshot the apo-model after 80 ns simulation. Key residues identified by mutagenesis are highlighted as stick representations. Schematic interaction maps of the docked ligand, generated via MOE with an interaction cut-off of 6 Å are shown for the Inward (C) and Outward (D) models. Residues starred are conserved hydrophobic residues common to both the Inward and Outward ligand binding pockets. (E) Affinity of ucb 30889 for recombinant rat SV2A (wt and mutants). A concentration range of ucb 30889 was incubated with 5 nM of [^3^H]ucb 30889 during 120 min at 4°C. B0 is the binding of [^3^H]ucb 30889 in the absence of any competing compound. Data are representative of three independent experiments. pIC_50_ values were calculated from untransformed raw data by non-linear regression using a model describing a sigmoidal dose-response curve with variable slope and are reported in Table 3. The position of the mutants with respect to the ligand in the Outward model is shown in (F).

The binding cavity in the Inward and Outward models is pictured in Fig. 4A,B and schematically shown in Fig. 4C,D. Residues known to impact binding as reported previously by Shi et al. [26], were found to interact with the docked ligand: Y462, W666 and K694 for the Inward-ucb 30889 system and C297, W300, M301, Y462 and K694 for the Outward-ucb 30889 system. In the Inward model, C297 does appear to interact with the ligand, but is still in close proximity. Analysis of interaction time throughout the simulations between ligand and protein has further highlighted these residues and indicated those key in each system to be: Y462, W666, N667 and K694 in the Inward-ucb 30889 and C297, W300, Y462, N667 and K694 in the Outward-ucb 30889 system (Fig. 4A, B). In addition to these residues, the Outward-ucb 30889 showed a key interaction between D670 and ucb 30889, showing that 2 of the 3 charged residues in the binding cavity are interacting with the ligand. Thus overall, the ligand-binding cavity is predicted to have a predominantly hydrophobic character supplemented by two or three polar residues; N667, K694 and D670 in the Outward model.

**Table 3:** Affinity of ucb 30889 for rat recombinant SV2A (wildtype and mutants) labeled with [^3^H]ucb 30889 (n = 3).

Rat recombinant SV2A	pIC_50_ (mean ± S.D.)
Wildtype	7.1 ± 0.05
W454A	6.7 ± 0.14
D670A	/
F688A	7.1 ± 0.19

The model allowed us to make predictions that could be tested experimentally—3 residues were explored; W454, F688 and D670. W454 is located near to the binding site, but in the Inward open model is pointing away from the binding cavity. In the Outward model, W454 does not appear to interact directly with ucb 30889 when docked to the last simulation frame, but it is however, pointing towards the cavity and potentially could interact with the ligand (Fig. 4F). Indeed, in MD simulations (Outward-ucb 30889), we found that the ligand interacts with W454 for 21% of the time (across all 3 repeats of Outward-ucb 30889). Thus we chose this residue to help delineate the two models better, and predicted that there would be a modest effect on ligand-binding for this residue. F688 is found at the cytosolic end of the TM cavity in the Inward open model and is buried in the Outward open model, and on this basis we predicted the mutation to have very little, if any, effect on the ligand binding site. D670, in the Inward-apo model, is located at the edge of the cavity, but in the Outward-apo model was located in a more central (in terms of the membrane normal) location and could potentially interact with K694. Indeed in the simulations, the distance between the carboxy oxygens of D670 and the amino hydrogens of K694 was less than 3.5 Å for 35% of the simulation time (with a mean of 4.5 ± 2.6 Å). Given the proposed transporter nature of SV2A, we hypothesized that this interaction may be necessary to help stabilize the Outward open conformation and thus replacing D670 with alanine should result in a decrease in binding ucb 30889. Thus, we predicted that mutating this residue would have a large impact on ligand-binding.

These predictions were borne out by experiments. As predicted, only a small effect on affinity was observed experimentally for W454A and there was almost no effect for F688A (Fig. 4E, Table 3). The position of the W454 is very different in the Inward open and Outward open models. In the Inward open model it is pointing away from the binding cavity, and although we cannot rule out indirect packing effects, we take this to suggest that the Outward open model accounts for this result better as in that model it does point into the cavity (Fig. 4F). For D670A the experiments again confirmed the prediction, with the binding being completely abolished in a radioligand binding assay (Fig. 4E). The position of the D670 in the Inward open model, although suggests it points towards the cavity, is nevertheless on the edge of the cavity. Given that the D670A mutant abolishes binding and the importance of interactions between ligand and TM helix 5 (W300A, C297alkylated are each reported to abolish binding by Shi et al. [26]), the position in the Outward open model is much more conducive to interaction with the ligand and thus we take this also to favour the Outward-apo model over the Inward-apo model. However, we must be extremely cautious, because mutations can manifest their influence on binding through indirect changes as well as direct changes. Indeed, this has been explicitly demonstrated for SV2 proteins where mutant proteins can for example become trapped in the endoplasmic reticulum, presumably reflecting a misfolded state [50]. Another caveat that we should raise at this point is that the mutations are performed in HEK cells and thus the influence of any vesicle proteins on drug binding will also be absent. We must also keep in mind that the sequence identity between the templates and SV2A is extremely low and the possibility of structural differences remains high at this level of similarity.

We should also be clear that we have generated two independent models here, rather than a single model that corresponds to two different states. Although the latter might ultimately be desirable to investigate state-dependent binding effects, we felt that generating the best model for each state independently was more useful at this stage. The development of a unified model is an ongoing area of research.

## Conclusions

In this paper we have used homology modelling based on templates corresponding to two different possible states of SV2A. Analysis of the sequence conservation of hydrophobic residues in SV2A in conjunction with additional structural templates has allowed us to identify additional residues that play distinct roles in ucb 30889 (and by inference, LEV) binding. MD simulations of the apo-system confirmed that the model was stable in the timescale of 80 ns but with substantial flexibility within the TM regions. The results suggest that the Outward model is more consistent with the experimental data than the Inward model, though we should stress caution there because the sequence identity between the templates and SV2A is very low. Nevertheless, we were able to use the models in a predictive way to advance our understanding of small-molecule SV2A interactions.

## Supporting Information

S1 Fig The consensus agreement for the position of α-helices (red) and β-sheets (blue) in SV2A, using HMMTop, PSIPred, SOSUI and JPRED. The 12 predicted TM helices for SV2 are indicated by black bars across the top of the alignment.
(PDF)

S2 Fig The final alignments for (A) GlpT and (B) FucP templates to SV2A, which were used to produce the model. (PDF)

S3 Fig The average confidence in model at each residue as given by QMEANlocalscore, as calculated by QMEANclust [48], for the (A) Inward and (B) Outward models respectively. The plots indicate high confidence in the TM helices in all models from MODELLER. Helices are indicated by black lines (standard deviation across the 100 models is shown in pale red)
(PDF)

S4 Fig The alignment of 24 sequences identified as SV2A in the uniprotKB database—red indicates complete conservation, blue similarity and grey greater variability. These indicate complete conservation of D670 and K694, while Y462 is found in all but one sequence (where it is glutamine).
(PDF)
