# Supplementary material for: Exploring the Interaction of SV2A with Racetams Using Homology Modelling, Molecular Dynamics and Site-Directed Mutagenesis
Source: PLoS One. 2015 Feb 18;10(2):e0116589. doi: 10.1371/journal.pone.0116589 (PMC4333566; doi:10.1371/journal.pone.0116589)
Supplement: S1 Fig — The 12 predicted TM helices for SV2 are indicated by black bars across the top of the alignment. (PDF) [file pone.0116589.s001.pdf]

Supporting Information for Lee et al.

SI Figure 1. The consensus agreement for the position of α-helices (red) and β-sheets (blue) in SV2A, using HMMTop, PSIPred, SOSUI and JPRED. The 12 predicted TM helices for SV2 are indicated by black bars across the top of the alignment.

```
HMMTop       MEEGFRDRAAFIRGAKDIAKEVKKHAAKKVVKGLDRVQDEYSRRSYSRFEEEEDDDDFPA  60
PSIPred      MEEGFRDRAAFIRGAKDIAKEVKKHAAKKVVKGLDRVQDEYSRRSYSRFEEEEDDDDFPA  60
SOSUI        MEEGFRDRAAFIRGAKDIAKEVKKHAAKKVVKGLDRVQDEYSRRSYSRFEEEEDDDDFPA  60
JPRED jnet   MEEGFRDRAAFIRGAKDIAKEVKKHAAKKVVKGLDRVQDEYSRRSYSRFEEEEDDDDFPA  60
JPRED jhmm   MEEGFRDRAAFIRGAKDIAKEVKKHAAKKVVKGLDRVQDEYSRRSYSRFEEEEDDDDFPA  60
JPRED jpssn  MEEGFRDRAAFIRGAKDIAKEVKKHAAKKVVKGLDRVQDEYSRRSYSRFEEEEDDDDFPA  60

HMMTop       PADGYYRGEGAQDEEEGGASSDATEGHDEDDEIYEGEYQGIPRAESGGKGERMADGAPLA 120
PSIPred      PADGYYRGEGAQDEEEGGASSDATEGHDEDDEIYEGEYQGIPRAESGGKGERMADGAPLA 120
SOSUI        PADGYYRGEGAQDEEEGGASSDATEGHDEDDEIYEGEYQGIPRAESGGKGERMADGAPLA 120
JPRED jnet   PADGYYRGEGAQDEEEGGASSDATEGHDEDDEIYEGEYQGIPRAESGGKGERMADGAPLA 120
JPRED jhmm   PADGYYRGEGAQDEEEGGASSDATEGHDEDDEIYEGEYQGIPRAESGGKGERMADGAPLA 120
JPRED jpssn  PADGYYRGEGAQDEEEGGASSDATEGHDEDDEIYEGEYQGIPRAESGGKGERMADGAPLA 120

HMMTop       GVRGGLSDGEGPPGGRGEAQRRKDREELAQQYETILRECGHGRFQWTLYFVLGLALMADG 180
PSIPred      GVRGGLSDGEGPPGGRGEAQRRKDREELAQQYETILRECGHGRFQWTLYFVLGLALMADG 180
SOSUI        GVRGGLSDGEGPPGGRGEAQRRKDREELAQQYETILRECGHGRFQWTLYFVLGLALMADG 180
JPRED jnet   GVRGGLSDGEGPPGGRGEAQRRKDREELAQQYETILRECGHGRFQWTLYFVLGLALMADG 180
JPRED jhmm   GVRGGLSDGEGPPGGRGEAQRRKDREELAQQYETILRECGHGRFQWTLYFVLGLALMADG 180
JPRED jpssn  GVRGGLSDGEGPPGGRGEAQRRKDREELAQQYETILRECGHGRFQWTLYFVLGLALMADG 180

HMMTop       VEVFVVGFVLPSAEKDMCLSDSNKGMLGLIVYLGMMVGAFLWGGLADRLGRRQCLLISLS 240
PSIPred      VEVFVVGFVLPSAEKDMCLSDSNKGMLGLIVYLGMMVGAFLWGGLADRLGRRQCLLISLS 240
SOSUI        VEVFVVGFVLPSAEKDMCLSDSNKGMLGLIVYLGMMVGAFLWGGLADRLGRRQCLLISLS 240
JPRED jnet   VEVFVVGFVLPSAEKDMCLSDSNKGMLGLIVYLGMMVGAFLWGGLADRLGRRQCLLISLS 240
JPRED jhmm   VEVFVVGFVLPSAEKDMCLSDSNKGMLGLIVYLGMMVGAFLWGGLADRLGRRQCLLISLS 240
JPRED jpssn  VEVFVVGFVLPSAEKDMCLSDSNKGMLGLIVYLGMMVGAFLWGGLADRLGRRQCLLISLS 240

HMMTop       VNSVFAFFSSFVQGYGTFLFCRLLSGVGIGGSIPIVFSYFSEFLAQEKRGEHLSWLCMFW 300
PSIPred      VNSVFAFFSSFVQGYGTFLFCRLLSGVGIGGSIPIVFSYFSEFLAQEKRGEHLSWLCMFW 300
SOSUI        VNSVFAFFSSFVQGYGTFLFCRLLSGVGIGGSIPIVFSYFSEFLAQEKRGEHLSWLCMFW 300
JPRED jnet   VNSVFAFFSSFVQGYGTFLFCRLLSGVGIGGSIPIVFSYFSEFLAQEKRGEHLSWLCMFW 300
JPRED jhmm   VNSVFAFFSSFVQGYGTFLFCRLLSGVGIGGSIPIVFSYFSEFLAQEKRGEHLSWLCMFW 300
JPRED jpssn  VNSVFAFFSSFVQGYGTFLFCRLLSGVGIGGSIPIVFSYFSEFLAQEKRGEHLSWLCMFW 300

HMMTop       MIGGVYAAAMAWAIIPHYGWSFQMGSAYQFHSWRVFVLVCAFPSVFAIGALTTQPESPRF 360
PSIPred      MIGGVYAAAMAWAIIPHYGWSFQMGSAYQFHSWRVFVLVCAFPSVFAIGALTTQPESPRF 360
SOSUI        MIGGVYAAAMAWAIIPHYGWSFQMGSAYQFHSWRVFVLVCAFPSVFAIGALTTQPESPRF 360
JPRED jnet   MIGGVYAAAMAWAIIPHYGWSFQMGSAYQFHSWRVFVLVCAFPSVFAIGALTTQPESPRF 360
JPRED jhmm   MIGGVYAAAMAWAIIPHYGWSFQMGSAYQFHSWRVFVLVCAFPSVFAIGALTTQPESPRF 360
JPRED jpssn  MIGGVYAAAMAWAIIPHYGWSFQMGSAYQFHSWRVFVLVCAFPSVFAIGALTTQPESPRF 360

HMMTop       FLENGKHDEAWMVLKQVHDTNMRAKGHPERVFSVTHIKTIHQEDELIEIQSDTGTWYQRW 420
PSIPred      FLENGKHDEAWMVLKQVHDTNMRAKGHPERVFSVTHIKTIHQEDELIEIQSDTGTWYQRW 420
SOSUI        FLENGKHDEAWMVLKQVHDTNMRAKGHPERVFSVTHIKTIHQEDELIEIQSDTGTWYQRW 420
JPRED jnet   FLENGKHDEAWMVLKQVHDTNMRAKGHPERVFSVTHIKTIHQEDELIEIQSDTGTWYQRW 420
JPRED jhmm   FLENGKHDEAWMVLKQVHDTNMRAKGHPERVFSVTHIKTIHQEDELIEIQSDTGTWYQRW 420
JPRED jpssn  FLENGKHDEAWMVLKQVHDTNMRAKGHPERVFSVTHIKTIHQEDELIEIQSDTGTWYQRW 420

HMMTop       GVRALSLGGQVWGNFLSCFSPEYRRITLMMMGVWFTMSFSYYGLTVWFPDMIRHLQAVDY 480
PSIPred      GVRALSLGGQVWGNFLSCFSPEYRRITLMMMGVWFTMSFSYYGLTVWFPDMIRHLQAVDY 480
SOSUI        GVRALSLGGQVWGNFLSCFSPEYRRITLMMMGVWFTMSFSYYGLTVWFPDMIRHLQAVDY 480
JPRED jnet   GVRALSLGGQVWGNFLSCFSPEYRRITLMMMGVWFTMSFSYYGLTVWFPDMIRHLQAVDY 480
JPRED jhmm   GVRALSLGGQVWGNFLSCFSPEYRRITLMMMGVWFTMSFSYYGLTVWFPDMIRHLQAVDY 480
JPRED jpssn  GVRALSLGGQVWGNFLSCFSPEYRRITLMMMGVWFTMSFSYYGLTVWFPDMIRHLQAVDY 480

HMMTop       AARTKVFPGERVEHVTFNFTLENQIHRGGQYFNDKFIGLRLKSVSFEDSLFEECYFEDVT 540
PSIPred      AARTKVFPGERVEHVTFNFTLENQIHRGGQYFNDKFIGLRLKSVSFEDSLFEECYFEDVT 540
```

```
SOSUI        AARTKVFPGERVEHVTFNFTLENQIHRGGQYFNDKFIGLRLKSVSFEDSLFEECYFEDVT 540
JPRED jnet   AARTKVFPGERVEHVTFNFTLENQIHRGGQYFNDKFIGLRLKSVSFEDSLFEECYFEDVT 540
JPRED jhmm   AARTKVFPGERVEHVTFNFTLENQIHRGGQYFNDKFIGLRLKSVSFEDSLFEECYFEDVT 540
JPRED jpssn  AARTKVFPGERVEHVTFNFTLENQIHRGGQYFNDKFIGLRLKSVSFEDSLFEECYFEDVT 540

HMMTop       SSNTFFRNCTFINTVFYNTDLFEYKFVNSRLVNSTFLHNKEGCPLDVTGTGEGAYMVYFV 600
PSIPred      SSNTFFRNCTFINTVFYNTDLFEYKFVNSRLVNSTFLHNKEGCPLDVTGTGEGAYMVYFV 600
SOSUI        SSNTFFRNCTFINTVFYNTDLFEYKFVNSRLVNSTFLHNKEGCPLDVTGTGEGAYMVYFV 600
JPRED jnet   SSNTFFRNCTFINTVFYNTDLFEYKFVNSRLVNSTFLHNKEGCPLDVTGTGEGAYMVYFV 600
JPRED jhmm   SSNTFFRNCTFINTVFYNTDLFEYKFVNSRLVNSTFLHNKEGCPLDVTGTGEGAYMVYFV 600
JPRED jpssn  SSNTFFRNCTFINTVFYNTDLFEYKFVNSRLVNSTFLHNKEGCPLDVTGTGEGAYMVYFV 600

HMMTop       SFLGTLAVLPGNIVSALLMDKIGRLRMLAGSSVLSCVSCFFLSFGNSESAMIALLCLFGG 660
PSIPred      SFLGTLAVLPGNIVSALLMDKIGRLRMLAGSSVLSCVSCFFLSFGNSESAMIALLCLFGG 660
SOSUI        SFLGTLAVLPGNIVSALLMDKIGRLRMLAGSSVLSCVSCFFLSFGNSESAMIALLCLFGG 660
JPRED jnet   SFLGTLAVLPGNIVSALLMDKIGRLRMLAGSSVLSCVSCFFLSFGNSESAMIALLCLFGG 660
JPRED jhmm   SFLGTLAVLPGNIVSALLMDKIGRLRMLAGSSVLSCVSCFFLSFGNSESAMIALLCLFGG 660
JPRED jpssn  SFLGTLAVLPGNIVSALLMDKIGRLRMLAGSSVLSCVSCFFLSFGNSESAMIALLCLFGG 660

HMMTop       VSIASWNALDVLTVELYPSDKRTTAFGFLNALCKLAAVLGISIFTSFVGITKAAPILFAS 720
PSIPred      VSIASWNALDVLTVELYPSDKRTTAFGFLNALCKLAAVLGISIFTSFVGITKAAPILFAS 720
SOSUI        VSIASWNALDVLTVELYPSDKRTTAFGFLNALCKLAAVLGISIFTSFVGITKAAPILFAS 720
JPRED jnet   VSIASWNALDVLTVELYPSDKRTTAFGFLNALCKLAAVLGISIFTSFVGITKAAPILFAS 720
JPRED jhmm   VSIASWNALDVLTVELYPSDKRTTAFGFLNALCKLAAVLGISIFTSFVGITKAAPILFAS 720
JPRED jpssn  VSIASWNALDVLTVELYPSDKRTTAFGFLNALCKLAAVLGISIFTSFVGITKAAPILFAS 720

HMMTop       AALALGSSLALKLPETRGQVLQ 742
PSIPred      AALALGSSLALKLPETRGQVLQ 742
SOSUI        AALALGSSLALKLPETRGQVLQ 742
JPRED jnet   AALALGSSLALKLPETRGQVLQ 742
JPRED jhmm   AALALGSSLALKLPETRGQVLQ 742
JPRED jpssn  AALALGSSLALKLPETRGQVLQ 742
```
