# Supplementary material for: Exploring the Interaction of SV2A with Racetams Using Homology Modelling, Molecular Dynamics and Site-Directed Mutagenesis
Source: PLoS One. 2015 Feb 18;10(2):e0116589. doi: 10.1371/journal.pone.0116589 (PMC4333566; doi:10.1371/journal.pone.0116589)
Supplement: S2 Fig — (PDF) [file pone.0116589.s002.pdf]

Supporting Information for Lee et al.

SI Figure 2. The final alignments for (A) GlpT and (B) FucP templates to SV2A, which were used to produce the model.

A)

```
>GlpT:1PW4A
----FKPAPHKARLPAAEIDPTYRRLRWQIFLGIFFGYAAYYLVRKNFALAMPYLVEQGF
S-----RGDLGFALSGISIAYGFSKFIMGSVSDRS---NPRVFLPAGLILAAAVMLFMGF
VPWAT-S------SIAVMFVLLFLCGWFQGMGWPPCGRTMVHWWS-QKE----RGGIVSV
WNCAHNVGGGIPPLLFLLG----------------------MAWFNDWHAALYMPAFCAI
LVALFAFAMMRDTPQSCGLPPIEEYKND--------------------------------
TAKQIFMQYVLPNKLLWYIAIANVFVYLLRYGILDWS------PTYLKEVKHFALDKSSW
AYFLYEYAGIPGTLLCGWMSDKVFR----GNRGATGVFFMTLVTIATIVYWMNP--AGNP
TVDMICMIVIGFLIYGPVMLIGLHALELAPK------KAAGTAAGFTGLFGYLGGSVAAS
AIVGYTVDFFGWDGGFMV-MIGGSILAVILLIVVMIGEKRRHEQLLQELVP----
>rSV2A
------------------VLGLALMADGVEVFVVGFVLPSAEKDMCLSDSNK--------
-------GMLGLIVYLGMMVGAFLWGGLADRLG--RRQCLLISLSVNSVFAFFSSFVQGY
GTFLF--------CRLLSGVGIGGSIPIVFSYFSEFLAQE---------KRGEHLSWLCM
FWMIGGVYAAAMAWAIIPHYGWSFQ-MGSAYQF--------------HSWRVFVLVCAFP
SVFAIGALTTQPESPRF-----------------------------------GGQVWGNF
LSCFSPEYRRITLMMMGVWFTMSFSYYGLTVWFPDMIRH----------------GEGAY
MVYFVSFLGTLAVLPGNIVSALLMD---KIGRLRMLAGSSVLSCVSCFFLSFGNSESA--
--MIALLCLFGGVSIASWNALDVLTVELYPSD-----K-RTTAFGFLNALCKLAAVLGIS
IFTSFVGI-------TKAAPILFASAALALGSSLALKLPETRGQVLQ--------
```

B)

```
>FucP:3O7PA
--RSYIIPFALLCSLFFLWAVANNLNDILLPQFQQAF--FQAGLIQSAFYFGYFIIPIP-
AGILMKKLSYKAGIITGLFLYALGAALFWPAAEIMNYTLFLVGLFIIAAGLGCLETAANP
FVTVLGPESSGHFRLNLAQTFASFGAIIAVVFGQSLILSNVPHQSQDVLDKMSPEQLSAY
KHSLVLSVQTPYMIIVAIVLLVALLIMLTKFPALQSDNHSDAKQGSFSASLSRLARIRHW
RWAVLAQFCYVGAQTACWSYLIRYAVEE------IPGMTAGFAANYLTGTMVCFFIGRFT
GTWLISRFAPHKVLAAYALIAMALCLISAFA------------GGHVGLIALTLCSAFMS
IQYPTIFSLGIKNLGQD-TKYGSSFIVMTIIGGGIVTPVMGFVSDAA----GNIPTAELI
PALCFAVIFIFARFRSQT---
>rSV2A
VLGLALMADGVEVFVVGFVLPSAEKDMCLSDSNK-----GMLGLIVYLGMMVGAFLWGGL
ADRLGRRQCLLISLSVNSVFAFFSSFVQGYGTFLFCRLLSGVGIGGSIPIVFSYFSEFLA
Q-------EKRGEHLSWLCMFWMIGGVYAAAMAWAIIPHYGWSFQMGSAYQF--------
----HSWRVFVLVCAFPSVFAIGALTTQPESPRF------GGQVWGNFLSCFSPEYRRIT
LMMMGVWFTMSFSYYGLTVWFPDMIRH-------------GEGAYMVYFVSFLGTLAVLP
GNIVSALLMDKIGRLRMLAGSSVLSCVSCFFLSFGNSES----AMIALLCLFGGVSIASW
NALDVLTVELYPSDKR--TTAFGFLNALCKLAAVLGISIFTSFVGI---TKAAPILFASA
ALALGSSLALKLPETRGQVLQ
```
