# Supplementary material for: Exploring the Interaction of SV2A with Racetams Using Homology Modelling, Molecular Dynamics and Site-Directed Mutagenesis
Source: PLoS One. 2015 Feb 18;10(2):e0116589. doi: 10.1371/journal.pone.0116589 (PMC4333566; doi:10.1371/journal.pone.0116589)

Supporting Information for Lee et al.

SI Figure 3. The average confidence in model at each residue as given by QMEANlocalscore, as calculated by QMEANclust[48], for the (A) Inward and (B) Outward models respectively. The plots indicate high confidence in the TM helices in all models from MODELLER. Helices are indicated by black lines (standard deviation across the 100 models is shown in pale red)

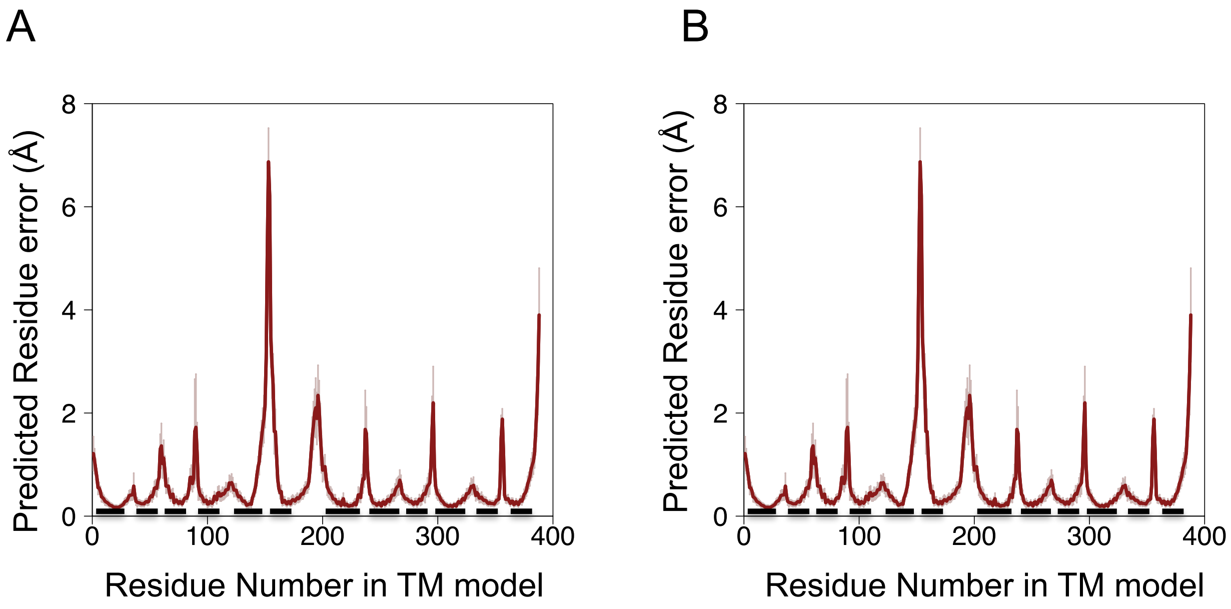

Supplement: S3 Fig — The plots indicate high confidence in the TM helices in all models from MODELLER. Helices are indicated by black lines (standard deviation across the 100 models is shown in pale red) (PDF) [file pone.0116589.s003.pdf]
