# Supplementary material for: Exploring the Interaction of SV2A with Racetams Using Homology Modelling, Molecular Dynamics and Site-Directed Mutagenesis
Source: PLoS One. 2015 Feb 18;10(2):e0116589. doi: 10.1371/journal.pone.0116589 (PMC4333566; doi:10.1371/journal.pone.0116589)
Supplement: S4 Fig — These indicate complete conservation of D670 and K694, while Y462 is found in all but one sequence (where it is glutamine). (PDF) [file pone.0116589.s004.pdf]

Supporting Information for Lee et al.

SI Figure 4. The alignment of 24 sequences identified as SV2A in the uniprotKB database – red indicates complete conservation, blue similarity and grey greater variability. These indicate complete conservation of D670 and K694, while Y462 is found in all but one sequence (where it is glutamine).

```
  sp|Q9JIS5|SV2A_MOUSE   -------MEEG--FRDRAAFIRGAKDIAKEVKKHAAKKVVKGLDRVQDEYSRRSYSRFEE 51
  sp|Q7L0J3|SV2A_HUMAN   -------MEEG--FRDRAAFIRGAKDIAKEVKKHAAKKVVKGLDRVQDEYSRRSYSRFEE 51
  sp|Q29397|SV2A_BOVIN   -------MEEG--FRDRAAFIRGAKDIAKEVKKHATKKVVKGLDRVQDEYSRRSYSRFEE 51
  sp|Q4R4X3|SV2A_MACFA   -------MEEG--FRDRAAFIRGAKDIAKEVKKHAAKKVVKGLDRVQDEYSRRSYSRFEE 51
  sp|Q5R4L9|SV2A_PONAB   -------MEEG--FRDRAAFIRGAKDIAKEVKKHAAKKVVKGLDRVQDEYSRRSYSRFEE 51
tr|F6QL25|F6QL25_XENTR   -------MEDT--YRDRTAFIRGAKDIAKEVKRHAAKKVGKGVDKMQDEYTKRSYTRFEE 51
tr|H2PZV7|H2PZV7_PANTR   -------MEEG--FRDRAAFIRGAKDIAKEVKKHAAKKVVKGLDRVQDEYSRRSYSRFEE 51
tr|G3VL92|G3VL92_SARHA   -------MDEG--FRDRTAFIRGAKDIAKEVKKHATKKVVKGLDRVQDEYSRRSYARFEE 51
tr|H2MNU2|H2MNU2_ORYLA   -LATSKMDDDGRYRDGRSDFIRGAKDIAKVAKKQVGKKVGRGVDKMADEYTRRSYKRFEE 59
tr|H2LD56|H2LD56_ORYLA   TVGDPFIMEDG--YQNRTAFIKGAKDIAKEVKRQASKKVGRSVDKMSDEYARRSYSRFEE 58
tr|H2SG74|H2SG74_TAKRU   -------MEDN--YQNRTAFIKGAKDIAKEVKRHASKKVGRTVDRMSDEYSMRSYSRFEE 51
tr|H2S661|H2S661_TAKRU   ------MDDDGRYRDGRSDFIRGAKDIAKVAKKQVGKKVGRGVDKMADEYTRRSYKRFEE 54
tr|D2I4U9|D2I4U9_AILME   -------MEEG--FRDRAAFIRGAKDIAKEVKKHAAKKVVKGLDRVQDEYSRRSYSRFEE 51
tr|M3WCP5|M3WCP5_FELCA   -------MEEG--FRDRAAFIRGAKDIAKEVKKHAAKKVVKGLDRVQDEYSRRSYSRFEE 51
tr|F7CQ51|F7CQ51_HORSE   -------MEEG--FRDRAAFIRGAKDIAKEVKKHAAKKVVKGLDRVQDEYSRRSYSRFEE 51
tr|F7DXP8|F7DXP8_MONDO   -------MDEG--FRDRTAFIRGAKDIAKEVKKHATKKVVKGLDRVQDEYSRRSYARFEE 51
tr|F1PFU3|F1PFU3_CANFA   -------MEEG--FRDRAAFIRGAKDIAKEVKKHAAKKVVKGLDRVQDEYSRRSYSRFEE 51
tr|A8K6Q3|A8K6Q3_HUMAN   -------MEEG--FRDRAAFIRGAKDIAKEVKKHAAKKVVKGLDRVQDEYSRRSYSRFEE 51
  tr|F1SDF9|F1SDF9_PIG   -------MEEG--FRDRAAFIRGAKDIAKEVKKHAAKKVVKGLDRVQDEYSRRSYSRFEE 51
tr|G1REY8|G1REY8_NOMLE   -------MEEG--FRDRAAFIRGAKDIAKEVKKHAAKKVVKGLDRVQDEYSRRSYSRFEE 51
tr|G1PGN6|G1PGN6_MYOLU   -------MEEG--FRDRAAFIRGAKDIAKEVKKHAAKKVGKGLDRVQDEYSRRSYSRFEE 51
tr|G1SKN4|G1SKN4_RABIT   -------MEEG--FRDRAAFIRGAKDIAKEVKKHATKKVVKGLDRVQDEYSRRSYSRFEE 51
tr|I3J3J0|I3J3J0_ORENI   -------MEDG--YQNRTAFIKGAKDIAKEVKRHAAKKVGRSVDRMSDEYSKRSYSRFEE 51
                         ============================================================
     sp|Q02563|SV2A_RAT   -------MEEG--FRDRAAFIRGAKDIAKEVKKHAAKKVVKGLDRVQDEYSRRSYSRFEE 51

  sp|Q9JIS5|SV2A_MOUSE   EDDDDDFPA---PADGYYRGEGAQD-EEEGGASSDATEGHDEDDEIYEGEYQGIPRAESG 107
  sp|Q7L0J3|SV2A_HUMAN   EDDDDDFPA---PSDGYYRGEGTQD-EEEGGASSDATEGHDEDDEIYEGEYQGIPRAESG 107
  sp|Q29397|SV2A_BOVIN   EDDDDDFPA---PADGYYRGEGAQD-EEEGGASSDATEGHDEDDEIYEGEYQGIPRAESG 107
  sp|Q4R4X3|SV2A_MACFA   EDDDDDFPA---PSDSYYRGEGTQD-EEEGGASSDATEGHDEDDEIYEGEYQGIPRAESG 107
  sp|Q5R4L9|SV2A_PONAB   EDDDDDFPA---PSDGYYRGEGTQD-EEEGGASSDATEGHDEDDEIYEGEYQGIPGAESG 107
tr|F6QL25|F6QL25_XENTR   DDDDD--YQ---PQDGYYRGDPLND---EEGASSDATEGHDEDDEIYEGEYQGIPRADSM 103
tr|H2PZV7|H2PZV7_PANTR   EDDDDDFPA---PSDGYYRGEGTQD-EEEGGASSDATEGHDEDDEIYEGEYQGIPRAESG 107
tr|G3VL92|G3VL92_SARHA   EDDDDDYPA---PADGYYRGDGVREDDEEGGASSDATEGHDEDEEIYEGEYQGIPRGESG 108
tr|H2MNU2|H2MNU2_ORYLA   EDDDDDYGGVPGGDSGYHRNDSRA--NDD-EGHSDSTEGHDEDDEIYEGEYQGIPRADSV 116
tr|H2LD56|H2LD56_ORYLA   DD-DDEYPMQGSQDGGYYRGDSTARNDDE-GGHSDSTEGHDEDDEIYEGEYQGIPRADSG 116
tr|H2SG74|H2SG74_TAKRU   DD-DDDYPVQGSQDGGYYRGDSQAANEDE-GG-SDSTEGHDEDDEIYEGEYQGIPRADSG 108
tr|H2S661|H2S661_TAKRU   EDDDDDYSGVPGNDDGYYRNDSRA--NDD-EGHSDSTEGHDEDDEIYEGEYQGIPRADSG 111
tr|D2I4U9|D2I4U9_AILME   EE-EEDFPA---PADGYYRGEGAQD-EEEGGASSDATEGHDEDDEIYEGEYQGIPRAESG 106
tr|M3WCP5|M3WCP5_FELCA   EEDDDDYPA---PADGYYRGEGAQD-EEEGGASSDATEGHDEDDEIYEGEYQGIPRAESG 107
tr|F7CQ51|F7CQ51_HORSE   EEDDDDFPA---PADGYYRGEGAQD-EEEAGASSDATEGHDEDDEIYEGEYQGIPRAESG 107
tr|F7DXP8|F7DXP8_MONDO   DDDDDDFPA---PADGYYRGEGVREDEEEGGASSDATEGHDEDEEIYEGEYQGIPRGESG 108
tr|F1PFU3|F1PFU3_CANFA   EEDDDDFPA---PADGYYHGEGAQD-EEEGGASSDATEGHDEDDEIYEGEYQGIPRAESG 107
tr|A8K6Q3|A8K6Q3_HUMAN   EDDDDDFPA---PSDGYYRGEGTQD-EEEGGASSDATEGHDEDDEIYEGEYQGIPRAESG 107
  tr|F1SDF9|F1SDF9_PIG   EEDDDDFPA---PADGYYRGEGAQD-EEEGGASSDATEGHDEDDEIYEGEYQGIPRAESG 107
tr|G1REY8|G1REY8_NOMLE   EDDDDDFPA---PSDGYYRGEGTQD-EEEGGASSDATEGHDEDDEIYEGEYQGIPRAESG 107
tr|G1PGN6|G1PGN6_MYOLU   DDDDDDYPA---PADGYYRGEGAQE-EEEGGVSSDATEGHDEDDEIYEGEYQGIPRAESG 107
tr|G1SKN4|G1SKN4_RABIT   EDDDDDYPA---PADGYYRGEGAQD-EEEGGASSDATEGHDEEDEIYEGEYQGIPRAESG 107
tr|I3J3J0|I3J3J0_ORENI   DD-DDDYPMQGSQDGGYYRGDSQAANDDE-GGHSDSTEGHDEDDEIYEGEYQGIPRADSG 109
                         ============================================================
     sp|Q02563|SV2A_RAT   EEDDDDFPA---PADGYYRGEGAQD-EEEGGASSDATEGHDEDDEIYEGEYQGIPRAESG 107

  sp|Q9JIS5|SV2A_MOUSE   GKGERMADGAPLAGVRGGLSDGEGPPGGRGEAQRRKDREELAQQYETILRECGHGRFQWT 167
  sp|Q7L0J3|SV2A_HUMAN   GKGERMADGAPLAGVRGGLSDGEGPPGGRGEAQRRKEREELAQQYEAILRECGHGRFQWT 167
  sp|Q29397|SV2A_BOVIN   GKGERMADGAPLAGVRGGLGDGEGPPGGRGEAQRRKEREELAQQYEAILRECGHGRFQWT 167
  sp|Q4R4X3|SV2A_MACFA   GKGERMADGAPLAGVRGGLSDGEGPPGGRGEAQRRKEREELAQQYEAILRECGHGRFQWT 167
  sp|Q5R4L9|SV2A_PONAB   GKGERMADGAPLAGVRGGLSDGEGPPGGRGEAQRRKEREELAQQYEAILRECSHGRFQWT 167
tr|F6QL25|F6QL25_XENTR   -KGDHLANNQQLVTEFKDF--------NDLEGQRKKDKEELAQQYEVILQECGHGRFQWT 154
tr|H2PZV7|H2PZV7_PANTR   GKGERMADGAPLAGVRGGLSDGEGPPGGRGEAQRRKEREELAQQYEAILRECGHGRFQWT 167
tr|G3VL92|G3VL92_SARHA   GKGERLADGAPLAGVRGGLGDGEGPG-IGGEAQRRKEREELAQQYETILRECGHGRFQWT 167
tr|H2MNU2|H2MNU2_ORYLA   KAG--SMDGVVAAQA-QQF-----RDLSAYEGERRKDQEELAQQYESILQECGHGKFQWT 168
tr|H2LD56|H2LD56_ORYLA   KGSLAGGPGSITAGA-QEF-----RDIDVSDAQRKKDKEELAQQYETILQECGHGRFQWS 170
tr|H2SG74|H2SG74_TAKRU   KGSLAGGPGSVKVDA-QQF-----RDIGASEAERRKDQEELAQQYETILQECGHGKFQWT 162
tr|H2S661|H2S661_TAKRU   KAP--GMDGVMTAEA-QQF-----RDLSAYEGERRKDQEELAQQYESILQECGHGKFQWT 163
tr|D2I4U9|D2I4U9_AILME   SKGERMADGAPLAGGRGGLGHGEGPPGGRGEAQRRKEREELAQQYEAILRECGHGRFQWT 166
tr|M3WCP5|M3WCP5_FELCA   GKGERMADGAPLAGVRGGLGDGEGPPGGRGEAQRRKEREELAQQYEAILRECGHGRFQWT 167
tr|F7CQ51|F7CQ51_HORSE   GKGERMVDGTPLAGVRGGLSDGEGPPGGRGEAQRRKEREELAQQYEAILRECGHGRFQWT 167
tr|F7DXP8|F7DXP8_MONDO   GKGDRLADGAPLAGVRGGLGDGEGPG-VGGEAQRRKEREELAQQYETILRECGHGRFQWT 167
tr|F1PFU3|F1PFU3_CANFA   GKGEQMADGATLAGVRGGLGDGEGPPGGRGEAQRRKEREELAQQYEAILRECGHGRFQWT 167
tr|A8K6Q3|A8K6Q3_HUMAN   GKGERMADGAPLAGVRGGLSDGEGPPGGRGEAQRRKEREELAQQYEAILRECGHGRFQWT 167
  tr|F1SDF9|F1SDF9_PIG   GKGERMADGAPLAGVRGGLGDGEGPPGGRGEAQRRKEREELAQQYETILRECGHGRFQWT 167
```

```
tr|G1REY8|G1REY8_NOMLE   GKGERMADGAPLAGVRGGLSDGEGPPGGRGEAQRRKEREELAQQYEAILRECGHGRFQWT 167
tr|G1PGN6|G1PGN6_MYOLU   GKGEQMADGAPLAGVRGGLDDGEGPPGGRGAAQRRKDREELAQQYETILRECGHGRFQWT 167
tr|G1SKN4|G1SKN4_RABIT   GKGERLADGAPLAGVRGGLSDGEGPPGGRGEAQRRKEREELAQQYEAILRECGHGRFQWT 167
tr|I3J3J0|I3J3J0_ORENI   KGSLAGGPGSVGAGA-QQF-----RDISVSEAERRKDQEELAQQYETILQECGHGKFQWS 163
                         ============================================================
     sp|Q02563|SV2A_RAT   GKGERMADGAPLAGVRGGLSDGEGPPGGRGEAQRRKDREELAQQYETILRECGHGRFQWT 167

    sp|Q9JIS5|SV2A_MOUSE   LYFVLGLALMADGVEVFVVGFVLPSAEKDMCLSDSNKGMLGLIVYLGMMVGAFLWGGLAD 227
    sp|Q7L0J3|SV2A_HUMAN   LYFVLGLALMADGVEVFVVGFVLPSAEKDMCLSDSNKGMLGLIVYLGMMVGAFLWGGLAD 227
    sp|Q29397|SV2A_BOVIN   LYFVLGLALMADGVEVFVVGFVLPSAEKDMCLSDSNKGMLGLIVYLGMMVGAFLWGGLAD 227
    sp|Q4R4X3|SV2A_MACFA   LYFVLGLALMADGVEVFVVGFVLPSAEKDMCLSDSNKGMLGLIVYLGMMVGAFLWGGLAD 227
    sp|Q5R4L9|SV2A_PONAB   LYFVLGLALMADGVEVFVVGFVLPSAEKDMCLSDSNKGMLGLIVYLGMMVGAFLWGGLAD 227
tr|F6QL25|F6QL25_XENTR   LYFVLGLALMADGVEIFVVGFVLPSAEKDMCLSDSNKGMLGLIVYLGMMVGAFLWGGMAD 214
tr|H2PZV7|H2PZV7_PANTR   LYFVLGLALMADGVEVFVVGFVLPSAEKDMCLSDSNKGMLGLIVYLGMMVGAFLWGGLAD 227
tr|G3VL92|G3VL92_SARHA   LYFVLGLALMADGVEVFVVGFVLPSAEKDMCLSDSNKGMLGLIVYLGMMVGAFLWGGLAD 227
tr|H2MNU2|H2MNU2_ORYLA   LYFVLGLALMADGVEIFVVGFVLPSAEKDMCLSEPNKGMLGLIVYLGMMVGAFVWGGLAD 228
tr|H2LD56|H2LD56_ORYLA   LYFVLGLALMADGVEIFVVGFVLPSAEKDMCLSEPNKSMLGLIVYFGMMVGAFLWGALAD 230
tr|H2SG74|H2SG74_TAKRU   LYFVLGLALMADGVEIFVVGFVLPSAEKDMCLSEPNKSMLGLIVYFGMMVGAFLWGALSD 222
tr|H2S661|H2S661_TAKRU   LYFVLGLALMADGVEIFVVGFVLPFAEKDMCLSEPNKGMLGLIVYLGMMVGAFVWGGLAD 223
tr|D2I4U9|D2I4U9_AILME   LYFVLGLALMADGVEVFVVGFVLPSAEKDMCLSDSNKGMLGLIVYLGMMVGAFLWGGLAD 226
tr|M3WCP5|M3WCP5_FELCA   LYFVLGLALMADGVEVFVVGFVLPSAEKDMCLSDSNKGMLGLIVYLGMMVGAFLWGGLAD 227
tr|F7CQ51|F7CQ51_HORSE   LYFVLGLALMADGVEVFVVGFVLPSAEKDMCLSDSNKGMLGLIVYLGMMVGAFLWGGLAD 227
tr|F7DXP8|F7DXP8_MONDO   LYFVLGLALMADGVEVFVVGFVLPSAEKDMCLSDSNKGMLGLIVYLGMMVGAFLWGGLAD 227
tr|F1PFU3|F1PFU3_CANFA   LYFVLGLALMADGVEVFVVGFVLPSAEKDMCLSDSNKGMLGLIVYLGMMVGAFLWGGLAD 227
tr|A8K6Q3|A8K6Q3_HUMAN   LYFVLGLALMADGVEVFVVGFVLPSAEKDMCLSDSNKGMLGLIVYLGMMVGAFLWGGLAD 227
  tr|F1SDF9|F1SDF9_PIG   LYFVLGLALMADGVEVFVVGFVLPSAEKDMCLSDSNKGMLGLIVYLGMMVGAFLWGGLAD 227
tr|G1REY8|G1REY8_NOMLE   LYFVLGLALMADGVEVFVVGFVLPSAEKDMCLSDSNKGMLGLIVYLGMMVGAFLWGGLAD 227
tr|G1PGN6|G1PGN6_MYOLU   LYFVLGLALMADGVEVFVVGFVLPSAEKDMCLSDSNKGMLGLIVYLGMMVGAFLWGGLAD 227
tr|G1SKN4|G1SKN4_RABIT   LYFVLGLALMADGVEIFVVGFVLPSAEKDMCLSDSNKGMLGLIVYLGMMVGAFLWGGLAD 227
tr|I3J3J0|I3J3J0_ORENI   LYFVLGLALMADGVEIFVVGFVLPSAEKDMCLSEPNKSMLGLIVYFGMMVGAFLWGALAD 223
                         ============================================================
     sp|Q02563|SV2A_RAT   LYFVLGLALMADGVEVFVVGFVLPSAEKDMCLSDSNKGMLGLIVYLGMMVGAFLWGGLAD 227

    sp|Q9JIS5|SV2A_MOUSE   RLGRRQCLLISLSVNSVFAFFSSFVQGYGTFLFCRLLSGVGIGGSIPIVFSYFSEFLAQE 287
    sp|Q7L0J3|SV2A_HUMAN   RLGRRQCLLISLSVNSVFAFFSSFVQGYGTFLFCRLLSGVGIGGSIPIVFSYFSEFLAQE 287
    sp|Q29397|SV2A_BOVIN   RLGRRQCLLISLSVNSVFAFFSSFVQGYGTFLFCRLLSGVGIGGSIPIVFSYFSEFLAQE 287
    sp|Q4R4X3|SV2A_MACFA   RLGRRQCLLISLSVNSVFAFFSSFVQGYGTFLFCRLLSGVGIGGSIPIVFSYFSEFLAQE 287
    sp|Q5R4L9|SV2A_PONAB   RLGRRQCLLISLSVNSVFAFFSSFVQGYGTFLFCRLLSGVGIGGSIPIVFSYFSEFLAQE 287
tr|F6QL25|F6QL25_XENTR   RIGRRQCLLISLSVNSVFAFFSSFVQGYGTFLFCRLLSGVGIGGSIPIVFSYFSEFLAQE 274
tr|H2PZV7|H2PZV7_PANTR   RLGRRQCLLISLSVNSVFAFFSSFVQGYGTFLFCRLLSGVGIGGSIPIVFSYFSEFLAQE 287
tr|G3VL92|G3VL92_SARHA   RLGRRQCLLISLSVNSVFAFFSSFVQGYGTFLLCRLLSGVGIGGSIPIVFSYFSEFLAQE 287
tr|H2MNU2|H2MNU2_ORYLA   RIGRRQTLLISLSINSVFAFFSSFVQGYSSFLFCRLASGVGIGGSIPIVFSYYSEFLAQE 288
tr|H2LD56|H2LD56_ORYLA   RIGRRQSLLICLSINSVFSFFSSFVQGYSTFLFCRLLSGVGIGGSIPIVFSYYSEFLAQE 290
tr|H2SG74|H2SG74_TAKRU   RIGRRQSLLICLSINSVFSFFSSFVQGYSTFLFCRLLSGVGIGGSIPIVFSYYSEFLAQE 282
tr|H2S661|H2S661_TAKRU   RIGRRQTLLIALSINSVFAFFSSFVQGYISFLFCRLTSGVGIGGSIPIVFSYYSEFLAQE 283
tr|D2I4U9|D2I4U9_AILME   RLGRRQCLLISLSVNSVFAFFSSFVQGYGTFLFCRLLSGVGIGGSIPIVFSYFSEFLAQE 286
tr|M3WCP5|M3WCP5_FELCA   RLGRRQCLLISLSVNSVFAFFSSFVQGYGTFLFCRLLSGVGIGGSIPIVFSYFSEFLAQE 287
tr|F7CQ51|F7CQ51_HORSE   RLGRRQCLLISLSVNSVFAFFSSFVQGYGTFLFCRLLSGVGIGGSIPIVFSYFSEFLAQE 287
tr|F7DXP8|F7DXP8_MONDO   RLGRRQCLLISLSVNSVFAFFSSFVQGYGTFLLCRLLSGVGIGGSIPIVFSYFSEFLAQE 287
tr|F1PFU3|F1PFU3_CANFA   RLGRRQCLLISLSVNSVFAFFSSFVQGYGTFLFCRLLSGVGIGGSIPIVFSYFSEFLAQE 287
tr|A8K6Q3|A8K6Q3_HUMAN   RLGRRQCLLISLSVNSVFAFFSSFVQGYGTFLFCRLLSGVGIGGSIPIVFSYFSEFLAQE 287
  tr|F1SDF9|F1SDF9_PIG   RLGRRQCLLISLSVNSVFAFFSSFVQGYGTFLFCRLLSGVGIGGSIPIVFSYFSEFLAQE 287
tr|G1REY8|G1REY8_NOMLE   RLGRRQCLLISLSVNSVFAFFSSFVQGYGTFLFCRLLSGVGIGGSIPIVFSYFSEFLAQE 287
tr|G1PGN6|G1PGN6_MYOLU   RLGRRQCLLISLSVNSVFAFFSSFVQGYGTFLFCRLLSGVGIGGSIPIVFSYFSEFLAQE 287
tr|G1SKN4|G1SKN4_RABIT   RLGRRQCLLISLSVNSVFAFFSSFVQGYGTFLFCRLLSGVGIGGSIPIVFSYFSEFLAQE 287
tr|I3J3J0|I3J3J0_ORENI   RIGRRQSLLISLSINSIFSFFSSFVQGYSTFLFCRLLSGVGIGGSIPIVFSYYSEFLSQE 283
                         ============================================================
     sp|Q02563|SV2A_RAT   RLGRRQCLLISLSVNSVFAFFSSFVQGYGTFLFCRLLSGVGIGGSIPIVFSYFSEFLAQE 287

    sp|Q9JIS5|SV2A_MOUSE   KRGEHLSWLCMFWMIGGVYAAAMAWAIIPHYGWSFQMGSAYQFHSWRVFVLVCAFPSVFA 347
    sp|Q7L0J3|SV2A_HUMAN   KRGEHLSWLCMFWMIGGVYAAAMAWAIIPHYGWSFQMGSAYQFHSWRVFVLVCAFPSVFA 347
    sp|Q29397|SV2A_BOVIN   KRGEHLSWLCMFWMIGGVYAAAMAWAIIPHYGWSFQMGSAYQFHSWRVFVLVCAFPSVFA 347
    sp|Q4R4X3|SV2A_MACFA   KRGEHLSWLCMFWMIGGVYAAAMAWAIIPHYGWSFQMGSAYQFHSWRVFVLVCAFPSVFA 347
    sp|Q5R4L9|SV2A_PONAB   KRGEHLSWLCMFWMIGGVYAAAMAWAIIPHYGWSFQMGSAYQFHSWRVFVLVCAFPSVFA 347
tr|F6QL25|F6QL25_XENTR   KRGEHLSWLCMFWMIGGIYASAMAWAIIPHYGWSFQMGSAYQFHSWRVFVLVCAFPSVFA 334
tr|H2PZV7|H2PZV7_PANTR   KRGEHLSWLCMFWMIGGVYAAAMAWAIIPHYGWSFQMGSAYQFHSWRVFVLVCAFPSVFA 347
tr|G3VL92|G3VL92_SARHA   KRGEHLSWLCMFWMIGGVYAAAMAWAIIPHYGWSFQMGSAYQFHSWRVFVLVCAFPSVFA 347
tr|H2MNU2|H2MNU2_ORYLA   KRGEHLSWLCMFWMIGGIYASAMAWAIIPHYGWSFQMGSAYQFHSWRVFVLVCAFPSVAA 348
tr|H2LD56|H2LD56_ORYLA   KRGEHLSWLCMFWMIGGIYASAMAWTIIPHYGWSFQMGSAYQFHSWRVFVLVCAFPSVAA 350
tr|H2SG74|H2SG74_TAKRU   KRGEHLSWLCMFWMMGGIYASAMAWAIIPHYGWSFQMGSAYQFHSWRVFVLVCAFPCVAA 342
tr|H2S661|H2S661_TAKRU   KRGEHLSWLCMFWMLGGIYAAAMAWAIIPHYGWSFQMGSAYQFHSWRVFVLVCAFPAVAA 343
tr|D2I4U9|D2I4U9_AILME   KRGEHLSWLCMFWMIGGVYAAAMAWAIIPHYGWSFQMGSAYQFHSWRVFVLVCAFPSVFA 346
tr|M3WCP5|M3WCP5_FELCA   KRGEHLSWLCMFWMIGGVYAAAMAWAIIPHYGWSFQMGSAYQFHSWRVFVLVCAFPSVFA 347
tr|F7CQ51|F7CQ51_HORSE   KRGEHLSWLCMFWMIGGVYAAAMAWAIIPHYGWSFQMGSAYQFHSWRVFVLVCAFPSVFA 347
tr|F7DXP8|F7DXP8_MONDO   KRGEHLSWLCMFWMIGGVYAAAMAWAIIPHYGWSFQMGSAYQFHSWRVFVLVCAFPSVFA 347
tr|F1PFU3|F1PFU3_CANFA   KRGEHLSWLCMFWMIGGVYAAAMAWAIIPHYGWSFQMGSAYQFHSWRVFVLVCAFPSVFA 347
tr|A8K6Q3|A8K6Q3_HUMAN   KRGEHLSWLCMFWMIGGVYAAAMAWAIIPHYGWSFQMGSAYQFHSWRVFVLVCAFPSVFA 347
  tr|F1SDF9|F1SDF9_PIG   KRGEHLSWLCMFWMIGGVYAAAMAWAIIPHYGWSFQMGSAYQFHSWRVFVLVCAFPSVFA 347
tr|G1REY8|G1REY8_NOMLE   KRGEHLSWLCMFWMIGGVYAAAMAWAIIPHYGWSFQMGSAYQFHSWRVFVLVCAFPSVFA 347
tr|G1PGN6|G1PGN6_MYOLU   KRGEHLSWLCMFWMIGGVYAAAMAWAIIPHYGWSFQMGSAYQFHSWRVFVLVCAFPSVFA 347
tr|G1SKN4|G1SKN4_RABIT   KRGEHLSWLCMFWMIGGVYAAAMAWAIIPHYGWSFQMGSAYQFHSWRVFVLVCAFPSVFA 347
tr|I3J3J0|I3J3J0_ORENI   KRGEHLSWLCMFWMIGGIYASAMAWAIIPHYGWSFQMGSAYQFHSWRVFVLVCAFPSVAA 343
                         ============================================================
     sp|Q02563|SV2A_RAT   KRGEHLSWLCMFWMIGGVYAAAMAWAIIPHYGWSFQMGSAYQFHSWRVFVLVCAFPSVFA 347
```

```
sp|Q9JIS5|SV2A_MOUSE     IGALTTQPESPRFFLENGKHDEAWMVLKQVHDTNMRAKGHPERVFSVTHIKTIHQEDELI 407
sp|Q7L0J3|SV2A_HUMAN     IGALTTQPESPRFFLENGKHDEAWMVLKQVHDTNMRAKGHPERVFSVTHIKTIHQEDELI 407
sp|Q29397|SV2A_BOVIN     IGALTTQPESPRFFLENGKHDEAWMVLKQVHDTNMRAKGHPERVFSVTHIKTIHQEDELI 407
sp|Q4R4X3|SV2A_MACFA     IGALTTQPESPRFFLENGKHDEAWMVLKQVHDTNMRAKGHPERVFSVTHIKTIHQEDELI 407
sp|Q5R4L9|SV2A_PONAB     IGALTTQPESPRFFLENGKHDEAWMVLKQVHDTNMRAKGHPERVFSVTHIKTIHQEDELI 407
tr|F6QL25|F6QL25_XENTR   IGALTTMPESPRFFLENGKHDEAWMVLKQVHDTNMRAKGHPERVFSVTQIKTIKQDDELV 394
tr|H2PZV7|H2PZV7_PANTR   IGALTTQPESPRFFLENGKHDEAWMVLKQVHDTNMRAKGHPERVFSVTHIKTIHQEDELI 407
tr|G3VL92|G3VL92_SARHA   IGALTTQPESPRFFLENGKHDEAWMVLKQVHDTNMRAKGHPERVFSVTHIKTIRQEDELI 407
tr|H2MNU2|H2MNU2_ORYLA   ISALSTMPESPRFYLENGKHDEAWMILKQVHDTNMRAKGCPERVFSVTTIKTVKPMDELV 408
tr|H2LD56|H2LD56_ORYLA   IAALNAMPESPRFYLENGKHDEGWMILKQVHDTNMRAKGHPEKVFSVTTIKTVKPADELV 410
tr|H2SG74|H2SG74_TAKRU   IAALNSMPESPRFYLENGKHDEGWMILKQVHDTNMRAKGYPERVFSVTTIKTVKQIDELV 402
tr|H2S661|H2S661_TAKRU   ICALTTMPESPRFFLENGKHDEAWMILKQVHDTNMRAKGYPERVFSVTTIKTVKQMEDLV 403
tr|D2I4U9|D2I4U9_AILME   IGALTTQPESPRFFLENGKHDEAWMVLKQVHDTNMRAKGHPERVFSVTHIKTIHQEDELI 406
tr|M3WCP5|M3WCP5_FELCA   IGALTTQPESPRFFLENGKHDEAWMVLKQVHDTNMRAKGHPERVFSVTHIKTIHQEDELI 407
tr|F7CQ51|F7CQ51_HORSE   IGALTTQPESPRFFLENGKHDEAWMVLKQVHDTNMRAKGHPERVFSVTHIKTIHQEDELI 407
tr|F7DXP8|F7DXP8_MONDO   IGALTTQPESPRFFLENGKHDEAWMVLKQVHDTNMRAKGHPERVFSVTHIKTIRQEDELI 407
tr|F1PFU3|F1PFU3_CANFA   IGALTTQPESPRFFLENGKHDEAWMVLKQVHDTNMRAKGHPERVFSVTHIKTIHQEDELI 407
tr|A8K6Q3|A8K6Q3_HUMAN   IGALTTQPESPRFFLENGKHDEAWMVLKQVHDTNMRAKGHPERVFSVTHIKTIHQEDELI 407
tr|F1SDF9|F1SDF9_PIG     IGALTTQPESPRFFLENGKHDEAWMVLKQVHDTNMRAKGHPERVFSVTHIKTIHQEDELI 407
tr|G1REY8|G1REY8_NOMLE   IGALTTQPESPRFFLENGKHDEAWMVLKQVHDTNMRAKGHPERVFSVTHIKTIHQEDELI 407
tr|G1PGN6|G1PGN6_MYOLU   IGALTTQPESPRFFLENGKHDEAWMVLKQVHDTNMRAKGHPERVFSVTHIKTIHQEDELI 407
tr|G1SKN4|G1SKN4_RABIT   IGALTTQPESPRFFLENGKHDEAWMVLKQVHDTNMRAKGHPERVFSVTHIKTIHQEDELI 407
tr|I3J3J0|I3J3J0_ORENI   IAALNAMPESPRFYLENGKHDEGWMVLKQVHDTNMRAKGYPERVFSVTTIKTVKQMDELV 403
                         ============================================================
sp|Q02563|SV2A_RAT       IGALTTQPESPRFFLENGKHDEAWMVLKQVHDTNMRAKGHPERVFSVTHIKTIHQEDELI 407

sp|Q9JIS5|SV2A_MOUSE     EIQSDTGTWYQRWGVRALSLGGQVWGNFLSCFSPEYRRITLMMMGVWFTMSF---SYYGL 464
sp|Q7L0J3|SV2A_HUMAN     EIQSDTGTWYQRWGVRALSLGGQVWGNFLSCFGPEYRRITLMMMGVWFTMSF---SYYGL 464
sp|Q29397|SV2A_BOVIN     EIQSDTGAWYQRWGVRALSLGGQVWGNFLSCFGPEYRRITLMMMGVWFTMSF---SYYGL 464
sp|Q4R4X3|SV2A_MACFA     EIQSDTGTWYQRWGVRALSLGGQVWGNFLSCFGPEYRRITLMMMGVWFTMSF---SYYGL 464
sp|Q5R4L9|SV2A_PONAB     EIQSDTGTWYQRWGVRALSLGGQVWGNFLSCFGPEYRRITLMMMGVWFTMSF---SYYGL 464
tr|F6QL25|F6QL25_XENTR   EIQSDTGALHRRWMIKLLNLSQEVWANFHQCFAPEYRRITLMMMAVWFTMSF---SYYGL 451
tr|H2PZV7|H2PZV7_PANTR   EIQSDTGTWYQRWGVRALSLGGQVWGNFLSCFGPEYRRITLMMMGVWFTMSF---SYYGL 464
tr|G3VL92|G3VL92_SARHA   EIQSDTGTWYQRWGVRAMSLGGQVWGNFLSCFSPEYRRITLMMMGVWFTMSF---SYYGL 464
tr|H2MNU2|H2MNU2_ORYLA   NMGD-GAAWHAKWRIKLTTLFHQVWNNFLTIFNPEYRRTTYMMMAVWFSMSF---RYQLL 464
tr|H2LD56|H2LD56_ORYLA   NIGT-DTPVWERYRQKIMSLSKQIRKNLLACFSPEYKRTTFMLMAVWFTMSF---SYYGL 466
tr|H2SG74|H2SG74_TAKRU   DIGT-DTPIWHRYRLKIMSLSQQIRKNIIACFSPEYKRTTFMLMAVWFTMSF---SYYGL 458
tr|H2S661|H2S661_TAKRU   NLGD-GAAWHEKWRIKLTTLFHQVWSNFQAVFSPEYRRTTYMMMAVWFSMSF---SYYGL 459
tr|D2I4U9|D2I4U9_AILME   EIQSDTGTWYQRWGVRALSLGGQVWGNFLSCFGPEYRRITLMMMGVWFTMSF---SYYGL 463
tr|M3WCP5|M3WCP5_FELCA   EIQSDTGTWYQRWGVRALSLGGQVWGNFLSCFGPEYRRITLMMMGVWFTMSF---SYYGL 464
tr|F7CQ51|F7CQ51_HORSE   EIQSDTGTWYQRWGVRALSLGGQVWRNFLSCFGPEYRRITLMMMGVWFTMSF---SYYGL 464
tr|F7DXP8|F7DXP8_MONDO   EIQSDTGTWYQRWGVRAMSLGGQVWGNFLSCFGPEYRRITLMMMGVWFTMSF---SYYGL 464
tr|F1PFU3|F1PFU3_CANFA   EIQSDTGTWYQRWGVRALSLGGQVWGNFLSCFGPEYRRITLMMMGVWFTMFLQVRSYYGL 467
tr|A8K6Q3|A8K6Q3_HUMAN   EIQSDTGTWYQRWGVRALSLGGQVWGNFLSCFGPEYRRITLMMMGVWFTMSF---SYYGL 464
tr|F1SDF9|F1SDF9_PIG     EIQSDTGTWYQRWGVRALSLGGQVWGNFLSCFGPEYRRITLMMMGVWFTMSF---SYYGL 464
tr|G1REY8|G1REY8_NOMLE   EIQSDTGTWYQRWGVRALSLGGQVWGNFLSCFGPEYRRITLMMMGVWFTMSF---SYYGL 464
tr|G1PGN6|G1PGN6_MYOLU   EIQSDTGTWYQRWGVRASNLGGQVWGNFLSCFGPEYRRITLMMMGVWFTMSF---SYYGL 464
tr|G1SKN4|G1SKN4_RABIT   EIQSDTGTWYQRWGVRALSLGGQVWGNFLSCFGPEYRRITLMMMGVWFTMSF---SYYGL 464
tr|I3J3J0|I3J3J0_ORENI   DVGT-DTPAWQRYRLKIMSLSQQIRNNILACFRPEYKRTTFMLMAVWFSMSF---SYYGL 459
                         ============================================================
sp|Q02563|SV2A_RAT       EIQSDTGTWYQRWGVRALSLGGQVWGNFLSCFSPEYRRITLMMMGVWFTMSF---SYYGL 464

sp|Q9JIS5|SV2A_MOUSE     TVWFPDMIR---------HLQAVDYAARTKVFPGERVEHVTFNFTLENQIHRGGQYFNDK 515
sp|Q7L0J3|SV2A_HUMAN     TVWFPDMIR---------HLQAVDYASRTKVFPGERVEHVTFNFTLENQIHRGGQYFNDK 515
sp|Q29397|SV2A_BOVIN     TVWFPDMIR---------HLQAVDYAARTKVFPGERVEHVTFNFTLENQIHRGGQYFNDK 515
sp|Q4R4X3|SV2A_MACFA     TVWFPDMIR---------HLQAVDYASRTKVFPGERVEHVTFNFTLENQIHRGGQYFNDK 515
sp|Q5R4L9|SV2A_PONAB     TVWFPDMIR---------HLQAVDYASRTKVFPGERVEHVTFNFTLENQIHRGGQYLNDK 515
tr|F6QL25|F6QL25_XENTR   TVWFPDMIK---------HLQNIDYASRTKYFHNESVNNFNFNFTLENQVHRKGEYHNDK 502
tr|H2PZV7|H2PZV7_PANTR   TVWFPDMIR---------HLQAVDYASRTKVFPGERVEHVTFNFTLENQIHRGGQYFNDK 515
tr|G3VL92|G3VL92_SARHA   TVWFPDMIR---------HLQAADYAARTRLFAGERVEHVTFNFTLENQIHRGGQYFNDK 515
tr|H2MNU2|H2MNU2_ORYLA   QDFLLSRTIKSRLFGLLVQLSKPSAENRSG-FLHCS-SKMESR--F-EHEHRRGLSYAFS 519
tr|H2LD56|H2LD56_ORYLA   TVWFPDMIK---------YIQKQEYDSRTKTFTKERVEHVTFNFTLENQIHRNGYYFNDK 517
tr|H2SG74|H2SG74_TAKRU   TVWFPDMIK---------YIQKQEYESRTKTFTNERVEHVTFNFTLENQVHRQGHYFNDK 509
tr|H2S661|H2S661_TAKRU   TVWFPDMIK---------YLQKQEYSSRTKVFIKEKVEHVTFNFTLENQVHRQGEYFNDK 510
tr|D2I4U9|D2I4U9_AILME   TVWFPDTIR---------HLQAVDYEARTKLFSGERVEHVTFNFTLENQIHRGGQYFNDK 514
tr|M3WCP5|M3WCP5_FELCA   TVWFPDMIR---------HLQAVDYEARTKLFSGEHVEHVTFNFTLENQIHRGGQYFNDK 515
tr|F7CQ51|F7CQ51_HORSE   TVWFPDMIR---------HLQAVDYAARTKVFPGERVEHVTFNFTLENQIHRGGQYFNDK 515
tr|F7DXP8|F7DXP8_MONDO   TVWFPDMIR---------HLQAVDYAARTRLFAGERVEHVTFNFTLENQIHRGGQYFNDK 515
tr|F1PFU3|F1PFU3_CANFA   TVWFPDMIR---------HLQAVDYEARTKLFSGERVEHVTFNFTLENQIHRGGQYFNDK 518
tr|A8K6Q3|A8K6Q3_HUMAN   TVWFPDMIR---------HLQAVDYASRTKVFPGERVEHVTFNFTLENQIHRGGQYFNDK 515
tr|F1SDF9|F1SDF9_PIG     TVWFPDMIR---------HLQAVDYAARTKVFPGERVEHVTFNFTLENQIHRGGQYFNDK 515
tr|G1REY8|G1REY8_NOMLE   TVWFPDMIR---------HLQAVDYASRTKVFPGERVEHVTFNFTLENQIHRGGQYFNDK 515
tr|G1PGN6|G1PGN6_MYOLU   TVWFPDMIR---------HLQAVDYAARTKVFPGERVEHVTFNFTLENQIHRGGQYFNDK 515
tr|G1SKN4|G1SKN4_RABIT   TVWFPDMIR---------HLQAVDYAARTKVFPGERVEHVTFNFTLENQIHRGGQYFNDK 515
tr|I3J3J0|I3J3J0_ORENI   TVWFPDMIK---------YIQKQEYESRTKTFTKERVEHVTFNFTLENQVHRQGYYFNDK 510
                         ============================================================
sp|Q02563|SV2A_RAT       TVWFPDMIR---------HLQAVDYAARTKVFPGERVEHVTFNFTLENQIHRGGQYFNDK 515

sp|Q9JIS5|SV2A_MOUSE     FIGLRLKSVSFEDSLFEECYFEDVTSSNTFFRNCTFINTVFYNTDLFEYKFVNSRLVNST 575
sp|Q7L0J3|SV2A_HUMAN     FIGLRLKSVSFEDSLFEECYFEDVTSSNTFFRNCTFINTVFYNTDLFEYKFVNSRLINST 575
sp|Q29397|SV2A_BOVIN     FIGLRLKSVSFEDSLFEECYFEDVTSSNTFFRNCTFINTVFYNTDLFEYKFVNSRLVNST 575
sp|Q4R4X3|SV2A_MACFA     FIGLRLKSVSFEDSLFEECYFEDVTSSNTFFRNCTFINTVFYNTDLFEYKFVNSRLVNST 575
sp|Q5R4L9|SV2A_PONAB     FIGLRLKSVSFEDSLFEECYFEDVTSSNTFFRNCTFINTVFYNTDLFEYKFVNSRLINST 575
```

```
tr|F6QL25|F6QL25_XENTR   FIGLKLKSVIFEDSLFTDCYFEDITSSNTFFKNCSFIRTMFYNTDLFDYKFINSKFTNST 562
tr|H2PZV7|H2PZV7_PANTR   FIGLRLKSVSFEDSLFEECYFEDITSSNTFFRNCTFINTVFYNTDLFEYKFVNSRLINST 575
tr|G3VL92|G3VL92_SARHA   FIGLRLKSVSFEDSLFEECYFEDVTSTNTFFRNCTFINTIFYNTDLFEYKFVGSRLINST 575
tr|H2MNU2|H2MNU2_ORYLA   --LVLIKYMILE--NWKPFFFLKI---SAAFYLCEVNISLIQQTHRILYRLINSRLINST 572
tr|H2LD56|H2LD56_ORYLA   FLNLKMKSMVFEDSVFEECYFEDITSTNTFFKNCTFIASLFYNTDLFKYRFVDCKLANST 577
tr|H2SG74|H2SG74_TAKRU   FLNLKMKYMVFEDSVFEECYFEDVTTTHTFFRNCTFIASLFYNTDLFKYRFVNCKLVNST 569
tr|H2S661|H2S661_TAKRU   FMNLKMRSMVFEDSLFEECFFEDITSSNTFFKNCTFIATLFYNTDLFKYRLVNCRLINST 570
tr|D2I4U9|D2I4U9_AILME   FIGLRLKSVSFEDSLFEECYFEDVTSSNTFFRNCTFINTVFYNTDLFEYKFVNSRLVNST 574
tr|M3WCP5|M3WCP5_FELCA   FIGLRLKSVSFEDSLFEECYFEDVTSSNTFFRNCTFINTVFYNTDLFEYKFVNSRLVNST 575
tr|F7CQ51|F7CQ51_HORSE   FIGLRLKSVSFEDSLFEECYFEDVTSSNTFFRNCTFINTVFYNTDLFEYKFVNSRLVNST 575
tr|F7DXP8|F7DXP8_MONDO   FIGLRLKSVSFEDSLFEECYFEDVTSSNTFFRNCTFINTIFYNTDLFEYKFVGSRLINST 575
tr|F1PFU3|F1PFU3_CANFA   FIGLRLKSVSFEDSLFEECYFEDVTSSNTFFRNCTFINTVFYNTDLFEYKFVNSRLVNST 578
tr|A8K6Q3|A8K6Q3_HUMAN   FIGLRLKSVSFEDSLFEECYFEDVTSSNTFFRNCTFIDTVFYNTDLFEYKFVNSRLINST 575
  tr|F1SDF9|F1SDF9_PIG   FIGLRLKSVSFEDSLFEECYFEDVTSSNTFFRNCTFINTVFYNTDLFEYKFVNSRLVNST 575
tr|G1REY8|G1REY8_NOMLE   FIGLRLKSVSFEDSLFEECYFEDVTSSNTFFRNCTFINTVFYNTDLFEYKFVNSRLINST 575
tr|G1PGN6|G1PGN6_MYOLU   FIGLRLKSVTFEDSLFEECYFEDVTSSNTFFRNCTFINTVFYNTDLFEYKFVNSHLVNST 575
tr|G1SKN4|G1SKN4_RABIT   FIGLRLKSVSFEDSLFEECYFEDVTSTNTFFRNCTFINTVFYNTDLFEYKFVNSRLVNST 575
tr|I3J3J0|I3J3J0_ORENI   FLNLKMKSMVFEDSVFEECYFEDITSTHTFFRNCTFIASLFYNTDLFKYRLVNCKLVNST 570
                         ============================================================
     sp|Q02563|SV2A_RAT   FIGLRLKSVSFEDSLFEECYFEDVTSSNTFFRNCTFINTVFYNTDLFEYKFVNSRLVNST 575

   sp|Q9JIS5|SV2A_MOUSE   FLHNKEGCPLDVTGTGEGAYMVYFVSFLGTLAVLPGNIVSALLMDKIGRLRMLAGSSVLS 635
   sp|Q7L0J3|SV2A_HUMAN   FLHNKEGCPLDVTGTGEGAYMVYFVSFLGTLAVLPGNIVSALLMDKIGRLRMLAGSSVMS 635
   sp|Q29397|SV2A_BOVIN   FLHNKEGCPLDVTGTGEGAYMVYFVSFLGTLAVLPGNIVSALLMDKIGRLRMLAGSSVMS 635
   sp|Q4R4X3|SV2A_MACFA   FLHNKEGCPLDVTGTGEGAYMVYFVSFLGTLAVLPGNIVSALLMDKIGRLRMLAGSSVMS 635
   sp|Q5R4L9|SV2A_PONAB   FLHNKEGCPLDVTGTGEGAYMVYFVSFLGTLAVLPGNIVSALLMDKIGRLRMLAGSSVMS 635
tr|F6QL25|F6QL25_XENTR   FLHSKEGCQLDFSDDINNAYMIYFVSFLGTLAVLPGNIVSALLMDKIGRLRMLAGSSVMS 622
tr|H2PZV7|H2PZV7_PANTR   FLHNKEGCPLDVTGTGEGAYMVYFVSFLGTLAVLPGNIVSALLMDKIGRLRMLAGSSVMS 635
tr|G3VL92|G3VL92_SARHA   FLHSKEGCPLDVTGTGEGAYMVYFVSFLGTLAVLPGNIVSALLMDKIGRLRMLAGSSVMS 635
tr|H2MNU2|H2MNU2_ORYLA   FLHNKEGCLLSDVSDENNAYMVYFVSFLGTLAVLPGNIVSALLMDKIGRLRMLAGSSVIS 632
tr|H2LD56|H2LD56_ORYLA   FLHNKEGCVLDFSDDFNNAYMIYFINFLGTLAVLPGNIVSALLMDKIGRLRMLAGSSIIS 637
tr|H2SG74|H2SG74_TAKRU   FLHNKEGCMLDFSDDFNNAYMIYFVNFLGTLAVLPGNIVSALLMDKIGRLRMLAGSSVIS 629
tr|H2S661|H2S661_TAKRU   FLHNKEGCMLSDVSDENNAYMVYFVSFLGTLAVLPGNIVSALLMDKIGRLRMLAGSSVIS 630
tr|D2I4U9|D2I4U9_AILME   FLHNKEGCPLDVTGTGEGAYMVYFVSFLGTLAVLPGNIVSALLMDKIGRLRMLAGSSVMS 634
tr|M3WCP5|M3WCP5_FELCA   FLHNKEGCPLDVTGTGEGAYMVYFVSFLGTLAVLPGNIVSALLMDKIGRLRMLAGSSVMS 635
tr|F7CQ51|F7CQ51_HORSE   FLHNKEGCPLDVTGTGEGAYMVYFVSFLGTLAVLPGNIVSALLMDKIGRLRMLAGSSVMS 635
tr|F7DXP8|F7DXP8_MONDO   FLHSKEGCPLDVAGTGEGAYMVYFVSFLGTLAVLPGNIVSALLMDKIGRLRMLAGSSVMS 635
tr|F1PFU3|F1PFU3_CANFA   FLHNKEGCPLDVTGTGEGAYMVYFVSFLGTLAVLPGNIVSALLMDKIGRLRMLAGSSVMS 638
tr|A8K6Q3|A8K6Q3_HUMAN   FLHNKEGCPLDVTGTGEGAYMVYFVSFLGTLAVLPGNIVSALLMDKIGRLRMLAGSSVMS 635
  tr|F1SDF9|F1SDF9_PIG   FLHNKEGCPLDVTGTGEGAYMVYFVSFLGTLAVLPGNIVSALLMDKIGRLRMLAGSSVMS 635
tr|G1REY8|G1REY8_NOMLE   FLHNKEGCPLDVTGTGEGAYMVYFVSFLGTLAVLPGNIVSALLMDKIGRLRMLAGSSVMS 635
tr|G1PGN6|G1PGN6_MYOLU   FLHNKEGCPLDVTGTGEGAYMVYFVSFLGTLAVLPGNIVSALLMDKIGRLRMLAGSSVMS 635
tr|G1SKN4|G1SKN4_RABIT   FLHNKEGCPLDVTGTGEGAYMVYFVSFLGSLAVLPGNIVSALLMDKIGRLRMLAGSSVMS 635
tr|I3J3J0|I3J3J0_ORENI   FLHNKEGCMLDFSDDFNNAYMIYFVNFLGTLAVLPGNIVSALLMDKIGRLRMLAGSSVIS 630
                         ============================================================
     sp|Q02563|SV2A_RAT   FLHNKEGCPLDVTGTGEGAYMVYFVSFLGTLAVLPGNIVSALLMDKIGRLRMLAGSSVLS 635

   sp|Q9JIS5|SV2A_MOUSE   CVSCFFLSFGNSESAMIALLCLFGGVSIASWNALDVLTVELYPSDKRTTAFGFLNALCKL 695
   sp|Q7L0J3|SV2A_HUMAN   CVSCFFLSFGNSESAMIALLCLFGGVSIASWNALDVLTVELYPSDKRTTAFGFLNALCKL 695
   sp|Q29397|SV2A_BOVIN   CVSCFFLSFGNSESAMIALLCLFGGVSIASWNALDVLTVELYPSDKRTTAFGFLNALCKL 695
   sp|Q4R4X3|SV2A_MACFA   CVSCFFLSFGNSESAMIALLCLFGGVSIASWNALDVLTVGLYPSDKRTTAFGFLNALCKL 695
   sp|Q5R4L9|SV2A_PONAB   CVSCFFLSFGNSESAMIALLCLFGGVSIASWNALDVLTVELYPSDKRTTAFGFLNALCKL 695
tr|F6QL25|F6QL25_XENTR   CISCFFLFFGNSESAMIALLCLFGGVSIASWNALDVLTVELYPSDKRTTAFGFLNALCKL 682
tr|H2PZV7|H2PZV7_PANTR   CVSCFFLSFGNSESAMIALLCLFGGVSIASWNALDVLTVELYPSDKRTTAFGFLNALCKL 695
tr|G3VL92|G3VL92_SARHA   CISCFFLSFGNSESAMIALLCLFGGVSIASWNALDVLTVELYPSDKRTTAFGFLNALCKL 695
tr|H2MNU2|H2MNU2_ORYLA   CISCFFLSFGNSESAMIALLCLFGGISIASWNALDVLTVELYPSDKRTTAFGFLNALCKL 692
tr|H2LD56|H2LD56_ORYLA   CVSCFFLMFGNSESGMIALLCLFGGISIASWNALDVVTVELYPSDKRTTAFGFLNALCKL 697
tr|H2SG74|H2SG74_TAKRU   CISCFFLMFGNSESGMIALLCLFGGISIASWNALDVITVELYPSDKRTTAFGFLNALCKL 689
tr|H2S661|H2S661_TAKRU   CISCFFLSFGNSESAMIALLCLFGGISIASWNSLDVLTVELYPSDKRTTAFGFLNALCKL 690
tr|D2I4U9|D2I4U9_AILME   CVSCFFLSFGNSESAMIALLCLFGGVSIASWNALDVLTVELYPSDKRTTAFGFLNALCKL 694
tr|M3WCP5|M3WCP5_FELCA   CVSCFFLSFGNSESAMIALLCLFGGVSIASWNALDVLTVELYPSDKRTTAFGFLNALCKL 695
tr|F7CQ51|F7CQ51_HORSE   CVSCFFLSFGNSESAMIALLCLFGGVSIASWNALDVLTVELYPSDKRTTAFGFLNALCKL 695
tr|F7DXP8|F7DXP8_MONDO   CISCFFLSFGNSESAMIALLCLFGGVSIASWNALDVLTVELYPSDKRTTAFGFLNALCKL 695
tr|F1PFU3|F1PFU3_CANFA   CVSCFFLSFGNSESAMIALLCLFGGVSIASWNALDVLTVELYPSDKRTTAFGFLNALCKL 698
tr|A8K6Q3|A8K6Q3_HUMAN   CVSCFFLSFGNSESAMIALLCLFGGVSIASWNALDVLTVELYPSDKRTTAFGFLNALCKL 695
  tr|F1SDF9|F1SDF9_PIG   CVSCFFLSFGNSESAMIALLCLFGGVSIASWNALDVLTVELYPSDKRTTAFGFLNALCKL 695
tr|G1REY8|G1REY8_NOMLE   CVSCFFLSFGNSESAMIALLCLFGGVSIASWNALDVLTVELYPSDKRTTAFGFLNALCKL 695
tr|G1PGN6|G1PGN6_MYOLU   CISCFFLSFGNSESAMIALLCLFGGVSIASWNALDVLTVELYPSDKRTTAFGFLNALCKL 695
tr|G1SKN4|G1SKN4_RABIT   CVSCFFLSFGNSESAMIALLCLFGGVSIASWNALDVLTVELYPSDKRTTAFGFLNALCKL 695
tr|I3J3J0|I3J3J0_ORENI   CVSCFFLMFGNTESGMIALLCLFGGISIASWNALDVITVELYPSDKRTTAFGFLNALCKL 690
                         ============================================================
     sp|Q02563|SV2A_RAT   CVSCFFLSFGNSESAMIALLCLFGGVSIASWNALDVLTVELYPSDKRTTAFGFLNALCKL 695

   sp|Q9JIS5|SV2A_MOUSE   AAVLGISIFTSFVGITKAAPILFASAALALGSSLALKLPETRGQVLQ 742
   sp|Q7L0J3|SV2A_HUMAN   AAVLGISIFTSFVGITKAAPILFASAALALGSSLALKLPETRGQVLQ 742
   sp|Q29397|SV2A_BOVIN   AAVLGISIFTSFVGITKAAPILFASAALALGSSLALKLPETRGQVLQ 742
   sp|Q4R4X3|SV2A_MACFA   AAVLGISIFTSFVGITKAAPIPFASAALALGSSLALKLPETRGQVLQ 742
   sp|Q5R4L9|SV2A_PONAB   AAVLGISIFTSFVGITKAAPILFASAALALGSSLALKLPETRGQVLQ 742
tr|F6QL25|F6QL25_XENTR   AAVLGISIFTSFVGVAKAVPILLASAALAVGSFLALKLPETRGQVLQ 729
tr|H2PZV7|H2PZV7_PANTR   AAVLGISIFTSFVGITKAAPILFASAALALGSSLALKLPETRGQVLQ 742
tr|G3VL92|G3VL92_SARHA   AAVLGISIFTSFVGVTKAAPILFASAALALGSSLALKLPETRGQVLQ 742
tr|H2MNU2|H2MNU2_ORYLA   AAVLGISIFTSFVGITKAVPILFASGALAVGSFLALKLPETRGQVLQ 739
tr|H2LD56|H2LD56_ORYLA   AAVLGISIFQSFVGITKALPILFAAGALAAGSFLATKLPETRGQVLQ 744
tr|H2SG74|H2SG74_TAKRU   AAVLGISIFQSFVGITKAVPIIFAAGALAAGSFLATKLPETRGQVLQ 736
```

```
tr|H2S661|H2S661_TAKRU  AAVLGISIFTSFVGITKAVPILFASGALAAGSFLALKLPETRGQVLK 737
tr|D2I4U9|D2I4U9_AILME  AAVLGISIFTSFVGITKAAPILFASAALALGSSLALKLPETRGQVLQ 741
tr|M3WCP5|M3WCP5_FELCA  AAVLGISIFTSFVGITKAAPILFASAALALGSSLALKLPETRGQVLQ 742
tr|F7CQ51|F7CQ51_HORSE  AAVLGISIFTSFVGITKAAPILFASAALALGSSLALKLPETRGQVLQ 742
tr|F7DXP8|F7DXP8_MONDO  AAVLGISIFTSFVGVTKAAPILFASAALALGSSLALKLPETRGQVLQ 742
tr|F1PFU3|F1PFU3_CANFA  AAVLGISIFTSFVGITKAAPILFASAALALGSSLALKLPETRGQVLQ 745
tr|A8K6Q3|A8K6Q3_HUMAN  AAVLGISIFTSFVGITKAAPILFASAALALGSSLALKLPETRGQVLQ 742
  tr|F1SDF9|F1SDF9_PIG  AAVLGISIFTSFVGITKAAPILFASAALALGSSLALKLPETRGQVLQ 742
tr|G1REY8|G1REY8_NOMLE  AAVLGISIFTSFVGITKAAPILFASAALALGSSLALKLPETRGQVLQ 742
tr|G1PGN6|G1PGN6_MYOLU  AAVLGISIFTSFVGITKAAPILFASAALALGSSLALKLPETRGQVLQ 742
tr|G1SKN4|G1SKN4_RABIT  AAVLGISIFTSFVGITKAAPILFASAALALGSSLALKLPETRGQVLQ 742
tr|I3J3J0|I3J3J0_ORENI  AAVLGISIFQSFVGITKAVPILFAAGALAAGSFLATKLPETRGQVLQ 737
                        ===============================================
    sp|Q02563|SV2A_RAT  AAVLGISIFTSFVGITKAAPILFASAALALGSSLALKLPETRGQVLQ 742
```
